# Diagnostic and Therapeutic Roles of Extracellular Vesicles in Chronic Kidney Disease: A Systematic Review

**DOI:** 10.1002/jev2.70300

**Published:** 2026-05-09

**Authors:** Tunahan Ergunay, Alessia Brossa, Benedetta Bussolati

**Affiliations:** ^1^ Department of Medical Sciences University of Turin Turin Italy; ^2^ Department of Molecular Biotechnology and Health Sciences University of Turin Turin Italy

**Keywords:** biological fluids, biological processes, diagnosis, exosomes, mesenchymal stromal cells, renal disease, therapy

## Abstract

Extracellular vesicles (EVs) are increasingly investigated across a wide range of diseases as potential biomarkers and therapeutic tools. To date, EVs have been isolated from diverse sources, including urine, blood, saliva, tissue, and cell cultures, with research focusing primarily on their protein and RNA cargo. Owing to their non‐invasive accessibility, selective cargo loading, and molecular richness, biological fluid–derived EVs have been proposed as promising candidates for biomarker discovery. In addition, several studies have explored EVs for therapeutic purposes, either by direct administration to diseased cells or organisms, by engineering them to enhance their efficacy, or by targeting them to modulate pathological processes. In this systematic review, we synthesize current evidence on the diagnostic and therapeutic roles of EVs in CKD and related conditions, integrating findings across different EV sources, cargos, and disease models, and providing an integrated perspective on the role of EVs in chronic kidney disease‐related research. By comparing molecular findings from diagnostic and therapeutic studies, we also identify key overlapping pathways and biological processes that may represent relevant mechanistic frameworks. Recognizing these convergent pathways can help unify data and guide future research toward mechanism‐driven, and clinically translatable EV applications in nephrology.

## Graphical abstract

This systematic review compiles and analyses 364 studies on chronic kidney disease (CKD) that investigated extracellular vesicles (EVs) for diagnostic and therapeutic applications. By systematically categorizing data according to EV source and cargo, it provides an integrated overview of EV application for CKD, highlighting common approaches and shared biological processes.

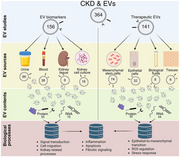

## 1 Introduction

Extracellular vesicles (EVs) are nanosized, lipid bilayer‐enclosed particles released by virtually all cell types, serving as fundamental mediators of intercellular communication through the transfer of bioactive molecules such as proteins, RNAs, and lipids. By modulating inflammation, fibrosis, and tissue repair, EVs have emerged as crucial players in both physiological homeostasis and pathological remodelling for tissue regeneration (Welsh et al. 2024). Chronic kidney disease (CKD), affecting nearly 10% of the global population, remains a major public health challenge associated with high morbidity, mortality, and socioeconomic burden. Current diagnostic methods typically detect kidney injury only after substantial functional decline, while available therapies predominantly slow, rather than reverse, disease progression (Bikbov et al. 2020). This unmet need has driven growing interest in EVs as accessible, mechanistically informative indicators of kidney health and disease (Zheng et al. 2024). EVs derived from kidney and non‐kidney cells circulate in various biofluids, including urine and plasma, providing minimally invasive means to monitor kidney pathology in real time. Increasing evidence implicates EVs in key processes underlying CKD and its complications, suggesting that their molecular cargo may serve as both diagnostic biomarkers and therapeutic targets. Furthermore, bioengineered EVs are being explored as versatile delivery vehicles for drugs, RNAs, and proteins, offering novel opportunities for targeted and personalized treatment approaches (Herrmann et al. 2021, Yang et al. 2024).

Despite these advances, the clinical translation of EV‐based applications is hindered by methodological heterogeneity in isolation, characterization, and study designs. In this systematic review, we synthesize current evidence on the diagnostic and therapeutic roles of EVs in CKD and related conditions, integrating findings across different EV sources, cargos, and disease models. By comparing molecular findings from diagnostic and therapeutic studies, we also identify key pathways and biological processes that may represent shared mechanistic frameworks. Recognizing these convergent pathways can help unify data and guide future research toward standardized, mechanism‐driven, and clinically translatable EV applications in nephrology.

## 2 Materials and Methods

### 2.1 Systematic Search

**FIGURE 1:**
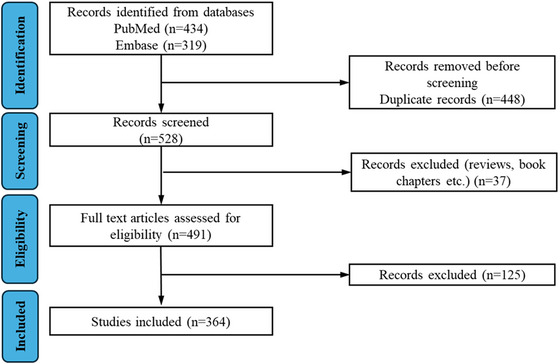
PRISMA flow diagram of the records included in the analysis of EV studies in CKD and related conditions.

We conducted a systematic search of PubMed and Embase databases up to May 22, 2025, following the Preferred Reporting Items for Systematic Reviews and Meta‐Analyses (PRISMA) guidelines (Figure 1). The search strategy included the terms “chronic kidney disease, CKD, kidney fibrosis, renal insufficiency, kidney failure, end‐stage renal disease, end‐stage kidney disease, ESRD, ESKD” and “extracellular vesicle, EV, exosome, microvesicle”. Other vesicle types such as microparticles and apoptotic bodies were not included in the search strategy as they represent distinct biological entities with different biogenesis mechanisms and were not consistently investigated in the context of CKD‐related EV research. Similarly, engineered nanoparticles or synthetic delivery systems were not included unless they were explicitly described as EVs or EV‐derived systems. The initial search yielded 434 records from PubMed and 319 from Embase. After removing duplicates, and excluding review articles, commentaries, book chapters, and unrelated studies such as studies that did not focus on CKD‐related diseases or did not study EVs, a total of 364 original research articles were included in the final analysis.

### 2.2 Categorization of Parameters

Studies were categorized according to their primary purpose as either diagnostic, focusing on biomarker discovery, or therapeutic, emphasizing potential clinical applications. Each study was further examined based on the disease, organism, EV source, and EV content. Studies that did not clearly fit into these categories, such as observational or mechanistic reports, were classified as “other”. In total, 156 studies were identified as diagnostic and 141 as therapeutic, with some overlapping in both categories.

In terms of analysed diseases, this review extended beyond CKD to include conditions closely linked to its development and progression. This encompassed acute kidney injury (AKI), AKD, the transition from AKI to CKD; kidney failure, the terminal stage of CKD; and diabetic kidney disease (DKD), one of the primary causes of kidney failure. Additionally, kidney fibrosis and kidney transplantation, although not distinct diseases, were included due to their direct relevance to CKD pathophysiology. Extra‐kidney and systemic disorders were also considered when strongly connected to kidney health. For instance, lupus, an immune‐mediated disease in which 10%–30% of patients progress to kidney failure, and cardiovascular diseases (CVD), including vascular calcification, which are highly prevalent in CKD and significantly increase mortality risk (Musa et al. 2025, Li and Lindholm 2023). Diseases with fewer than five records were grouped as “other diseases,” which included cirrhosis, sepsis, atherothrombosis, muscle atrophy, and renovascular diseases.

The organisms represented in these studies included human, mouse, rat, porcine, and cat models. To reduce ambiguity, human studies were further divided into “human (*in vitro*)” and “clinical.” For EV sources, umbrella terms were applied for clarity: kidney cell cultures (KCC) included proximal tubular cells and podocytes; “blood” encompassed both plasma and serum; and “mesenchymal stem cells (MSCs)” grouped studies using these cells. EV sources with fewer than 10 records were consolidated into an “other EV sources” category, which included amniotic fluid, aqueous humour, and macrophages. Regarding EV content, RNA was subdivided into messenger RNA (mRNA), microRNA (miRNA), circular RNA (circRNA), and long non‐coding RNA (lncRNA), while studies that employed total RNA sequencing were grouped under “total RNA.” A small number of studies investigated alternative EV contents such as lipids, cAMP, calcium, and polysaccharides, which were classified collectively as “other EV contents.”

### 2.3 Connecting the Parameters

To better illustrate the relationships between diseases, EV sources, and EV contents, we generated chord diagrams linking these parameters. These diagrams visualize how specific EV sources and contents are distributed across different diseases. The thickness of each connection reflects the relative number of studies within that category. In addition, the distribution of organism types studied under each category was included. It should be noted that some studies were classified under multiple categories—for example, a single study may be associated with both CKD and vascular calcification, or may include EVs derived from both blood and urine. Consequently, the total number of organisms represented does not necessarily correspond to the total number of records within each category.

### 2.4 Enrichment Analysis

**FIGURE 2:**
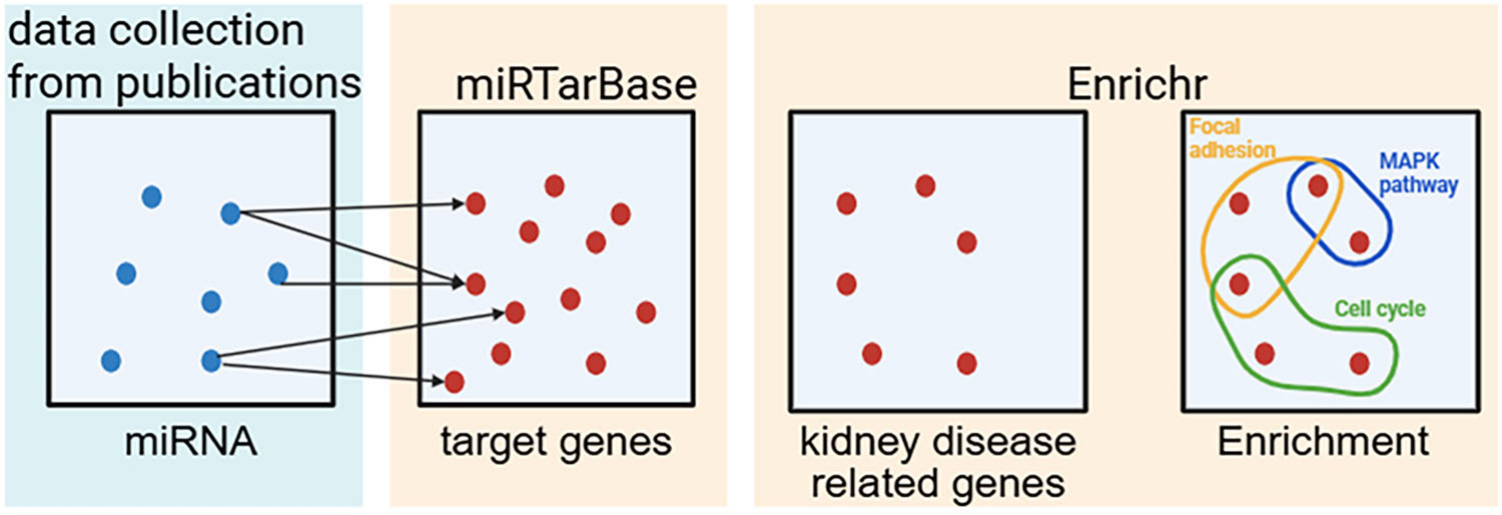
Data analysis workflow of miRNAs identified in therapeutic study records.

We collected proteins and miRNAs highlighted in the included studies and performed functional enrichment analysis. For miRNAs, their experimentally validated protein targets were identified using miRTarBase (Figure 2). To ensure disease relevance, proteins not associated with kidney‐related conditions were excluded through filtering with the DisGeNET database. The remaining proteins, together with those directly studied, were analysed for Gene Ontology (GO) Biological Process. Results were ranked by adjusted *p*‐values, and the key enriched terms related to kidney physiology and disease were presented.

## 3 Results and Discussion

**FIGURE 3:**
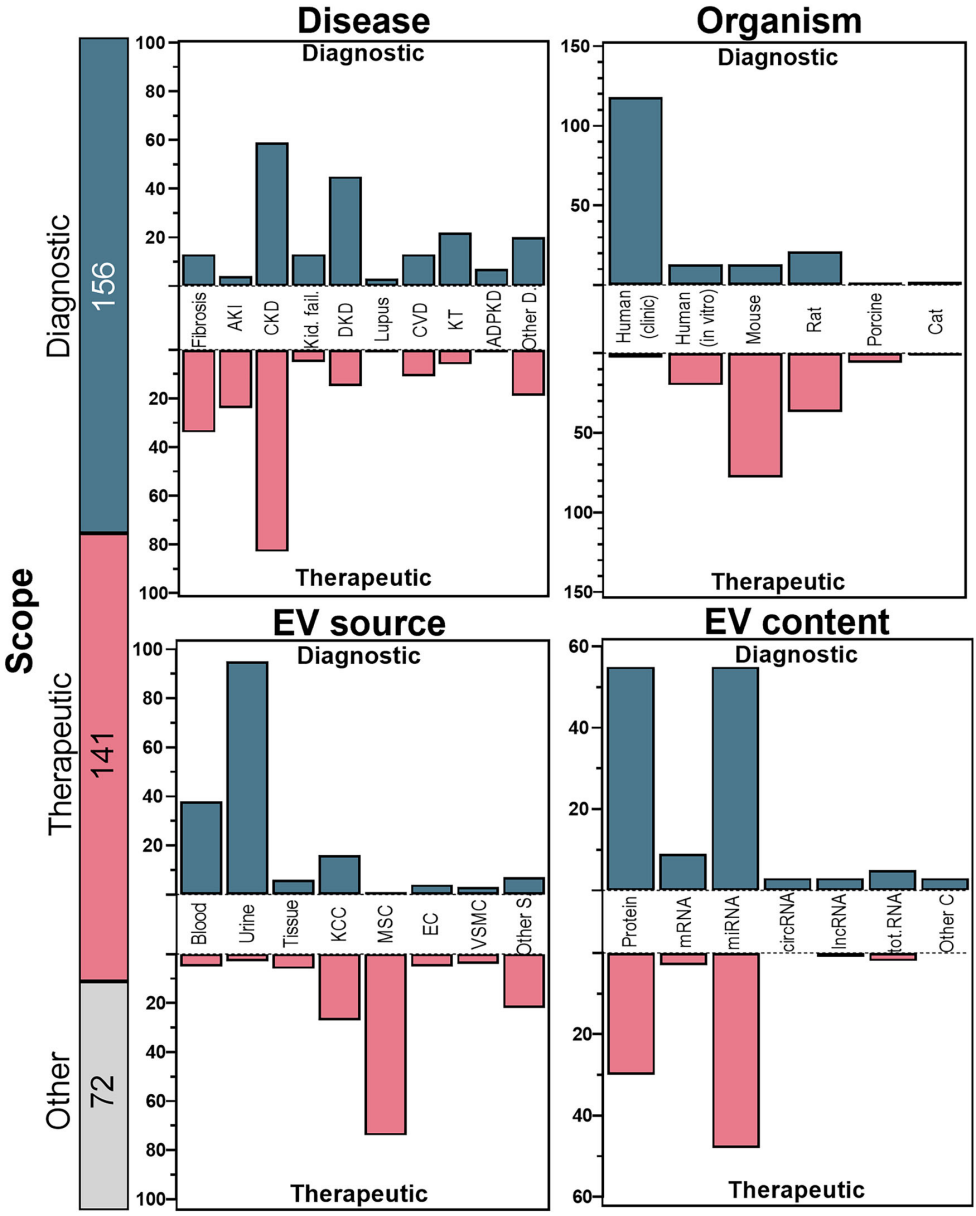
Categorisation of the records. Studies were subdivided according to their primary scope as diagnostic or therapeutic to obtain a clearer classification. The term organism in diagnostic studies refers to the EV origin, in therapeutic studies to the target organism. Studies primarily focused on mechanistic investigations, methodological advancements, or observational analyses that did not fit within diagnostic or therapeutic classifications were grouped under “other”. Fibrosis: kidney fibrosis, AKI: acute kidney injury, CKD: chronic kidney disease, kid. fail.: kidney failure, DKD: diabetic kidney disease, CVD: cardiovascular diseases, KT: kidney transplantation, ADPKD: autosomal dominant polycystic kidney disease, other D: other diseases, KCC: kidney cell culture, MSC: mesenchymal stem cell, EC: endothelial cell, VSMC: vascular smooth muscle cell, Other S: other EV sources, tot.RNA: total RNA screening, Other C: other EV contents.

EVs are emerging as dual‐purpose tools in CKD: minimally invasive diagnostic readouts and modular therapeutics. Across CKD and related conditions (AKI, DKD, kidney failure, fibrosis, transplantation), CKD itself was investigated in roughly equal numbers of diagnostic and therapeutic studies. Certain diseases showed a stronger emphasis on diagnostics, such as kidney failure, DKD, kidney transplantation, and ADPKD; in contrast, kidney fibrosis and AKI were more commonly explored in therapeutic studies (Figure 3). When categorizing by organism type, we found that human clinical studies dominated the diagnostic category, likely reflecting the accessibility of biological fluids such as blood and urine. Alongside clinical samples, diagnostic investigations also employed human *in vitro* systems and rodent models. Therapeutic studies, on the other hand, were conducted predominantly in mouse models, followed by human *in vitro* systems, rats, and porcine models. Importantly, only one therapeutic study used human clinical samples, underscoring the current gap in translating EV‐based therapeutic strategies into clinical practice. Studies primarily focused on mechanistic investigations, methodological developments, or observational analyses that do not directly test diagnostic biomarkers, or therapeutic interventions were grouped as “others”. In the context of this review, these studies may still provide critical mechanistic insights that support the identification of EV cargoes and pathways later explored for diagnostic or therapeutic applications.

In analysing the EV sources, we found that urine and blood were commonly utilized, primarily due to their abundance and ease of collection (Figure 3). These biofluids were predominantly used in diagnostic studies, reflecting the feasibility of employing EVs from accessible biological fluids for biomarker discovery. In contrast, EVs derived from MSCs and KCC were more frequently employed in therapeutic studies, due to their higher purity and the ability to control experimental variables and microenvironmental conditions. At present, there are no studies isolating EVs directly from kidney tissue, an interesting future approach for diagnostic information. At variance, few studies explored the diagnostic potential of EVs isolated from kidney‐derived or urine‐derived cells. In addition to these, endothelial cells and vascular smooth muscle cells (VSMCs) were also common sources of EVs. Less frequently, EVs were isolated from sources such as amniotic fluid, aqueous humour, bacteria, dental pulp stem cells, and macrophages—most of which were studied in a therapeutic context.

Regarding EV content, studies focusing on RNA and protein were similarly represented across the dataset (Figure 3). In diagnostic studies, proteins and miRNAs were the most analysed EV cargo. However, while proteins are more commonly assessed on diagnostic studies, miRNAs are slightly more investigated in therapeutic studies. This trend could be related to the concept that miRNA‐based interventions may offer longer‐lasting or wider regulation of disease‐associated gene expression. In addition to proteins and miRNAs, a smaller number of studies investigated other RNA species, including mRNA, circRNA, lncRNA, and total RNA. A limited number of studies also explored non‐RNA components such as lipids, cAMP, calcium, and polysaccharides as part of the EV cargo.

### 3.1 EVs as Diagnostic Biomarkers

#### EV Source: Urine

**FIGURE 4:**
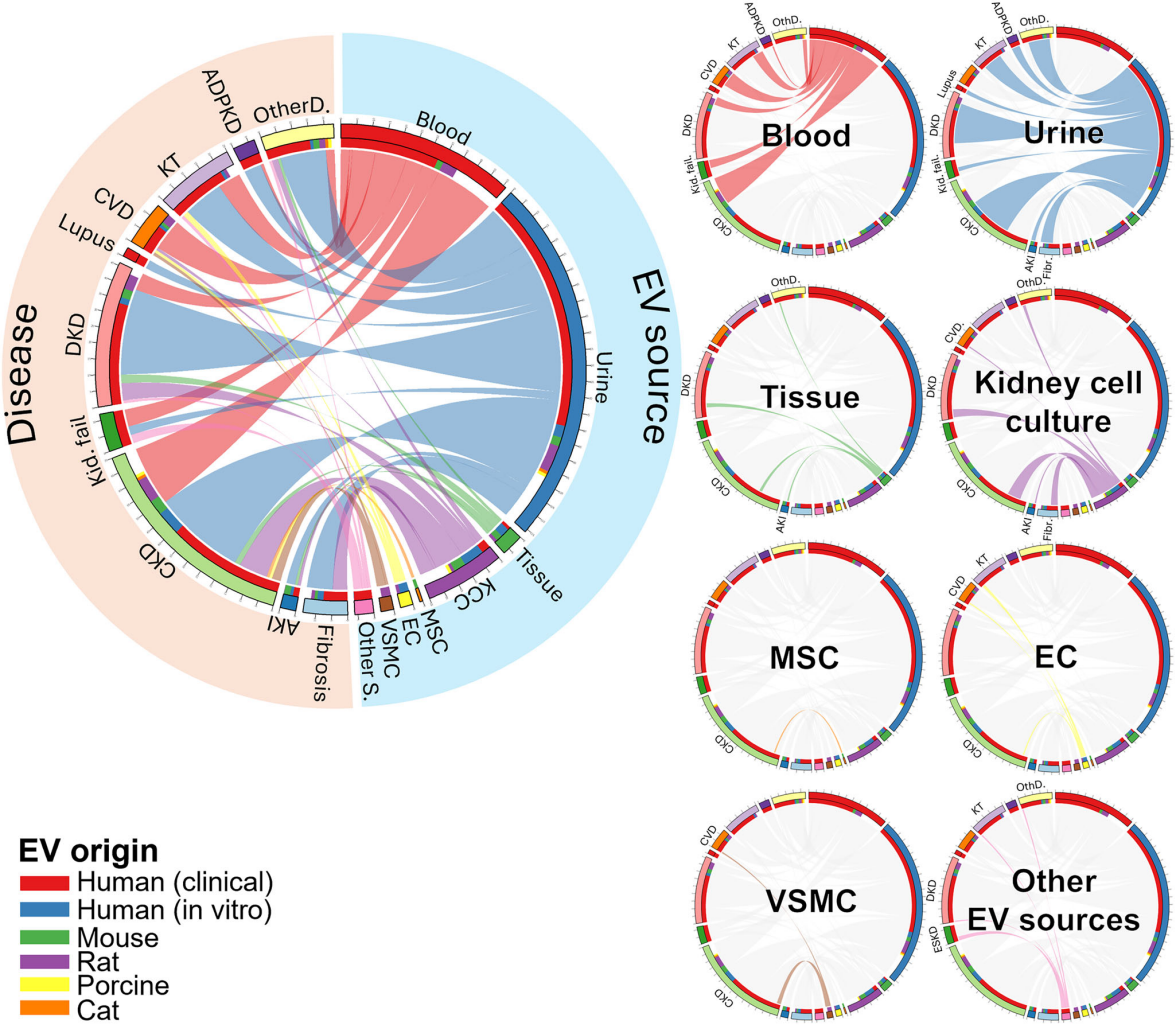
Chord diagram of diagnostic studies categorised by EV source. The outer layer represents EV sources and disease types, with links indicating the distribution of specific EV sources across diseases. The inner layer shows the EV originating organism (EV origin) used in each study. Ticks on the outer layer denote increments of five records. To facilitate interpretation, the distribution of individual EV sources is highlighted separately. KCC: kidney cell culture, MSC: mesenchymal stem cell, EC: endothelial cell, VSMC: vascular smooth muscle cell, Other S.: other EV sources, AKI: acute kidney injury, CKD: chronic kidney disease, Kid. fail.: kidney failure, DKD: diabetic kidney disease, CVD: cardiovascular diseases, KT: kidney transplantation, ADPKD: autosomal dominant polycystic kidney disease, OtherD: other diseases.

According to diagnostic studies, urine derived EVs are among the most frequently investigated sources for biomarker discovery (Figure 4). Urinary EVs offer a non‐invasive means to monitor disease status, reflect dynamic changes in molecular biomarkers, and can be obtained in relatively high quantities. In healthy individuals, urinary EVs primarily originate from epithelial cells lining the urogenital tract, as the glomerular filtration barrier (∼6 nm pore size) restricts the passage of larger vesicles (>20 nm) from systemic circulation (Svenningsen et al. 2020). This physiological compartmentalization makes urinary EVs particularly valuable for studying kidney disorders, as they provide site‐specific and disease‐specific information that directly reflects the cellular state within the nephron and urinary system.

There are several important factors to consider when studying urinary EVs. Since both EV concentration and cargo composition vary under different physiological and pathological conditions, the timing and type of urine collection become critical parameters in experimental design (Erdbrügger et al. 2021). Typically, the first morning void is preferred because it contains higher EV concentrations compared to other collections. However, it is advisable to examine potential EV biomarkers across multiple voids, as circadian variations can influence kidney function and EV release. Indeed, disruption of the circadian system has been associated with kidney fibrosis and the progression of CKD (Firsov and Bonny 2018). Some kidney‐related proteins and disease biomarkers display circadian‐dependent expression patterns, reflecting rhythmic kidney physiology. For instance, the first urine sample collected after digital rectal examination has been reported to contain a higher proportion of EVs associated with prostate cancer (McKiernan et al. 2016). Conversely, the first morning void tends to include greater contamination from cells and bacteria, which may interfere with EV isolation and downstream analyses (Manoni et al. 2012). A recent study monitoring the temporal dynamics of urinary EV excretion and cargo in healthy individuals revealed a diurnal pattern in NCC (sodium chloride cotransporter) expression during the day and an increase in AQP2 expression in the afternoon (Upson et al.). Although such studies have only recently begun to characterize these temporal patterns, further exploration of circadian effects on urinary EV biomarkers in kidney diseases would provide valuable insights.

#### EV Source: Blood

Blood‐derived EVs were also commonly utilized in diagnostic studies across nearly all disease categories (Figure 4). These EVs originate primarily from circulating cells such as erythrocytes, leukocytes, and platelets, and to a lesser extent from tissues and organs (Nieuwland and Siljander 2024). Researchers typically isolate EVs from either plasma or serum; however, several studies have highlighted compositional differences between these sources. In particular, serum‐derived EV preparations may lose larger or denser vesicles during the removal of blood cells, potentially resulting in underrepresentation of certain EV subpopulations. Consequently, some studies have suggested that plasma‐derived EVs more accurately reflect the circulating EV pool. Another critical consideration in blood‐derived EV studies is the tendency of platelets to become activated during sample handling, leading to artificial EV release and potentially confounding biomarker discovery. One comprehensive study investigated markers of endothelial damage in CKD patients with or without diabetes undergoing haemodialysis (Carmona et al. 2017). Authors reported a significant increase in plasma‐derived EVs, particularly large microvesicles, along with elevated CD14^+^/CD16^+^ monocytes and an increased Angiotensin 2/1 ratio in diabetic compared to nondiabetic patients. Importantly, plasma EV levels correlated with mortality risk, suggesting their potential as diagnostic markers for CVD in the context of CKD and diabetes. Similarly, another study investigated changes in plasma‐derived EVs in pediatric CKD patients and reported an increase in endothelial EV populations along with alterations in miRNA profiles, related to impaired angiogenesis and vascular pathology (Behrens et al. 2025). These findings further suggest that targeting EVs and their miRNA cargo may represent a potential therapeutic strategy to mitigate cardiovascular complications associated with CKD. Moreover, this study underscores the importance of EV profiling across different patient populations, including both adult and pediatric cohorts, to capture disease heterogeneity and age‐specific pathophysiological features.

#### EV Source: Tissue

Although EVs have not been directly isolated from affected kidney tissues, a number of studies correlated tissue expression profile with EV‐associated markers. Most of these investigations have focused on DKD, followed by CKD (Figure 4). For instance, the analysis of 250 microarray datasets of glomerular and tubulointerstitial tissues from patients with various CKD types, identified common differentially expressed genes (DEGs) (Zhou et al. 2018), significantly enriched in EV‐associated components. Similarly, another microarray study on glomerular tissues from DKD patients found a comparable enrichment of EV‐related DEGs (Zeng et al. 2019), suggesting their involvement in CKD and their role as diagnostic biomarkers.

Other studies investigated the impact of EVs derived from distant organs on kidney pathology. For example, one study investigated the role of bone marrow–derived EVs in kidney osteodystrophy, isolating EVs from the bone marrow of diseased rats and performing miRNA sequencing (Fu et al. 2023). Authors identified 24 differentially expressed miRNAs, involved in inflammation and osteoclast differentiation, which were proposed as contributors to the disease's pathogenesis. On a parallel perspective, a recent study showed that EVs released from CKD kidney tissue and detectable in the patient circulation may negatively impact the cardiac function (Li et al. 2026). The study identified several pro‐apoptotic miRNAs present in both human and mouse CKD‐derived EVs, functionally validated to induce cardiomyocyte apoptosis and cytotoxicity. These findings suggest that kidney‐released EVs during CKD may contribute to cardiotoxicity through the transfer of pro‐apoptotic miRNAs, which, in turn, could serve as potential early diagnostic biomarkers for reno‐cardiac interactions.

#### EV Source: Kidney Cell Culture

*In vitro* studies using KCC, represent the next most common area to discover diagnostic biomarkers (Figure 4), with a parallel effort to understand the mechanism of action of the candidate markers. For example, EVs isolated from tubular epithelial cells undergoing epithelial‐to‐mesenchymal transition (EMT) induced M1 polarization on macrophages (Lu et al. 2023), at variance of EVs under normal conditions. Moreover, injection of EVs from tubular cells undergoing EMT into mice caused an inflammatory response and M1 macrophage activation. In addition to tubular cells, podocytes, distal tubular cells, and other kidney cell types were also utilized. In different studies, the identification of biomarkers in kidney cell‐derived EVs was paralleled by their assessment in biological fluids, and vice versa. For instance, the levels of miR‐126 and miR‐145 in urinary EVs correlated with albuminuria in diabetic patients (Dimuccio et al. 2022, Barutta et al. 2022) and were paralleled by increased miR‐126 and miR‐145 in EVs from tubular cells and podocytes undergoing EMT (Dimuccio et al. 2022). Another study reported the presence of Elf3, an endothelium‐specific transcription factor, in damaged podocytes and their EVs (Sakurai et al. 2019). This was confirmed in the urine of patients with diabetic nephropathy, and suggested the existence of irreversible injuries in podocytes. Kidney cell culture models offer a controlled environment that enables more refined, mechanistic insights into EV biology, while also supporting animal‐free experimental approaches.

#### Other EV Sources

Other EV sources were less commonly used for diagnostic purposes (Figure 4). For instance, endothelial cell‐derived EVs were reported in only four studies, which focused on CKD, CVD, and kidney transplantation. Despite their rarity, these studies introduce novel approaches to EV‐based diagnostics. One notable example is a recent study investigating antenatal hydronephrosis, a condition that may lead to kidney failure in children and CKD in adults (Jin et al. 2021). Researchers isolated EVs from the amniotic fluid of ten pregnant women with grade III–IV antenatal hydronephrosis and performed transcriptomic sequencing. Among the six differentially expressed lncRNAs identified, LINC02863 was highlighted as a potential biomarker, due to its target genes' involvement in kidney development and morphogenesis.

Several studies used peritoneal dialysis (PD) effluent as a source of EVs to monitor ultrafiltration failure. One research group profiled the EV proteome in PD effluent from patients undergoing kidney replacement therapy and observed variability in the protein composition between newly enrolled and long‐term PD patients (Carreras‐Planella et al. 2017). In a subsequent longitudinal study, the same group monitored the EV profile of PD patients over a two‐year period and identified significant alterations in proteins, such as endoglin, that are associated with membrane instability and ultrafiltration loss (Carreras‐Planella et al. 2019). Another study examined the miRNA profile of EVs isolated from PD effluent of kidney insufficient patients experiencing ultrafiltration failure (Wu et al. 2022). Differentially expressed miRNAs were enriched in pathways related to ubiquitin‐mediated proteolysis, axon guidance, and Rap1 and Ras signalling, suggesting their potential involvement in PD‐associated membrane dysfunction.

#### EV Content

**FIGURE 5:**
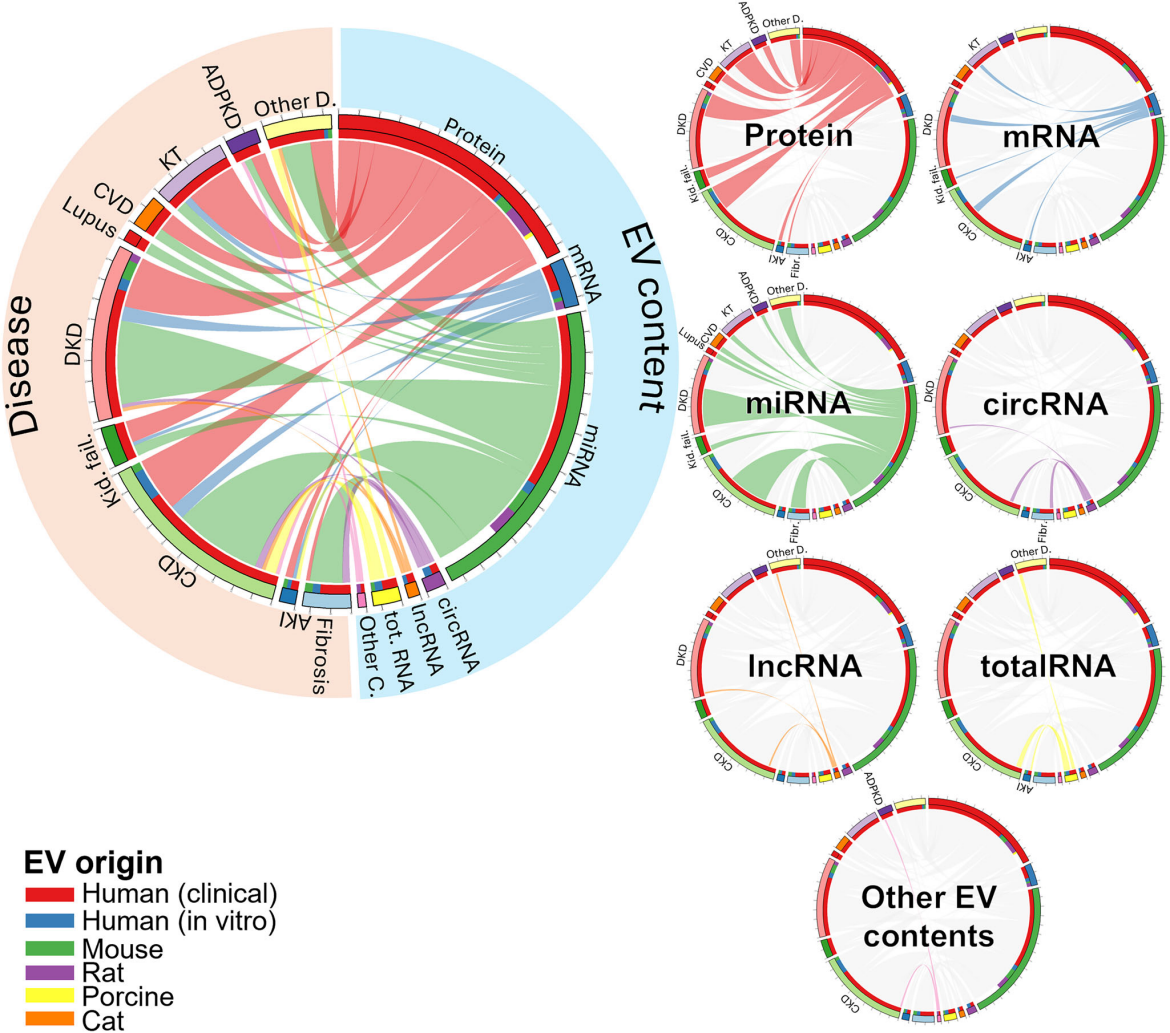
Chord diagram of diagnostic studies with EV content. The outer layer represents EV content and disease types, while the links indicate the distribution of specific EV content across diseases. The inner layer represents the EV originating organism (EV origin) used in the study. The ticks on the outer layer indicates every 5 records. To enhance clarity of links, EV contents were also highlighted individually. tot. RNA: total RNA, Other C: other EV contents, AKI: acute kidney injury, CKD: chronic kidney disease, Kid. fail.: kidney failure, DKD: diabetic kidney disease, CVD: cardiovascular diseases, KT: kidney transplantation, ADPKD: autosomal dominant polycystic kidney disease, OtherD: other diseases.

Given the diverse molecular composition of EVs, which varies depending on cell type, physiological state, and disease condition, EVs appear as a multifaced source of potential biomarkers. As previously noted, the majority of studies investigated proteins and miRNAs; however, other RNA species and molecular components were also explored (Figure 5).

#### EV Content: Proteins

Proteins were analysed across nearly all disease types (Figure 5). Due to the selective loading mechanisms involved in EV biogenesis, the protein content of EVs may differ from that of the origin cell or the corresponding biological fluid. In some cases, specific EV‐associated proteins have been proposed as diagnostic biomarkers. For example, in a proteomic analysis of urinary EVs from type 2 diabetic rat models, authors observed a significant increase in isocitrate dehydrogenase 1 (IDH1) levels during the early stages of disease (Sei et al. 2024). Importantly, IDH1 was not detected in EV‐free urine, suggesting that its enrichment is specific to EVs. The authors proposed that this increase may reflect pathophysiological changes in the proximal tubules, positioning EV‐associated IDH1 as a potential early biomarker for diabetic nephropathy, even prior to changes in albuminuria. In another study, the protein matrix remodelling–associated protein 5 (MXRA5) was evaluated as a non‐invasive biomarker in paediatric patients with uteropelvic junction obstruction (Wang et al. 2020). Although whole urine levels of MXRA5 did not differ significantly between patients and healthy controls, MXRA5 was markedly elevated in urinary EVs from affected individuals. These findings highlight the potential of EV‐associated MXRA5 as a disease‐specific biomarker for UPJO, which may not be detectable through conventional urine protein analysis.

While protein biomarker discovery studies are primarily conducted in humans, mice, and rats, one notable study utilized a porcine model to investigate the role of EVs in metabolic syndrome (Zhang et al. 2019). In this study, pigs were fed a high‐fat diet to induce metabolic syndrome and subsequently treated with elamipretide, a mitochondria‐targeted peptide. Authors observed elevated levels of nephrin and podocalyxin positive podocyte‐derived EVs in the urine of diseased pigs. These levels were significantly reduced following elamipretide treatment, suggesting a therapeutic effect. The findings were further supported by similar increases in podocyte‐derived urinary EVs observed in obese human patients, reinforcing the translational relevance of the pig model. Authors proposed that these EVs reflect early podocyte injury and mitochondrial dysfunction associated with the onset of metabolic syndrome and may serve as both early biomarkers and therapeutic targets in this context.

**FIGURE 6:**
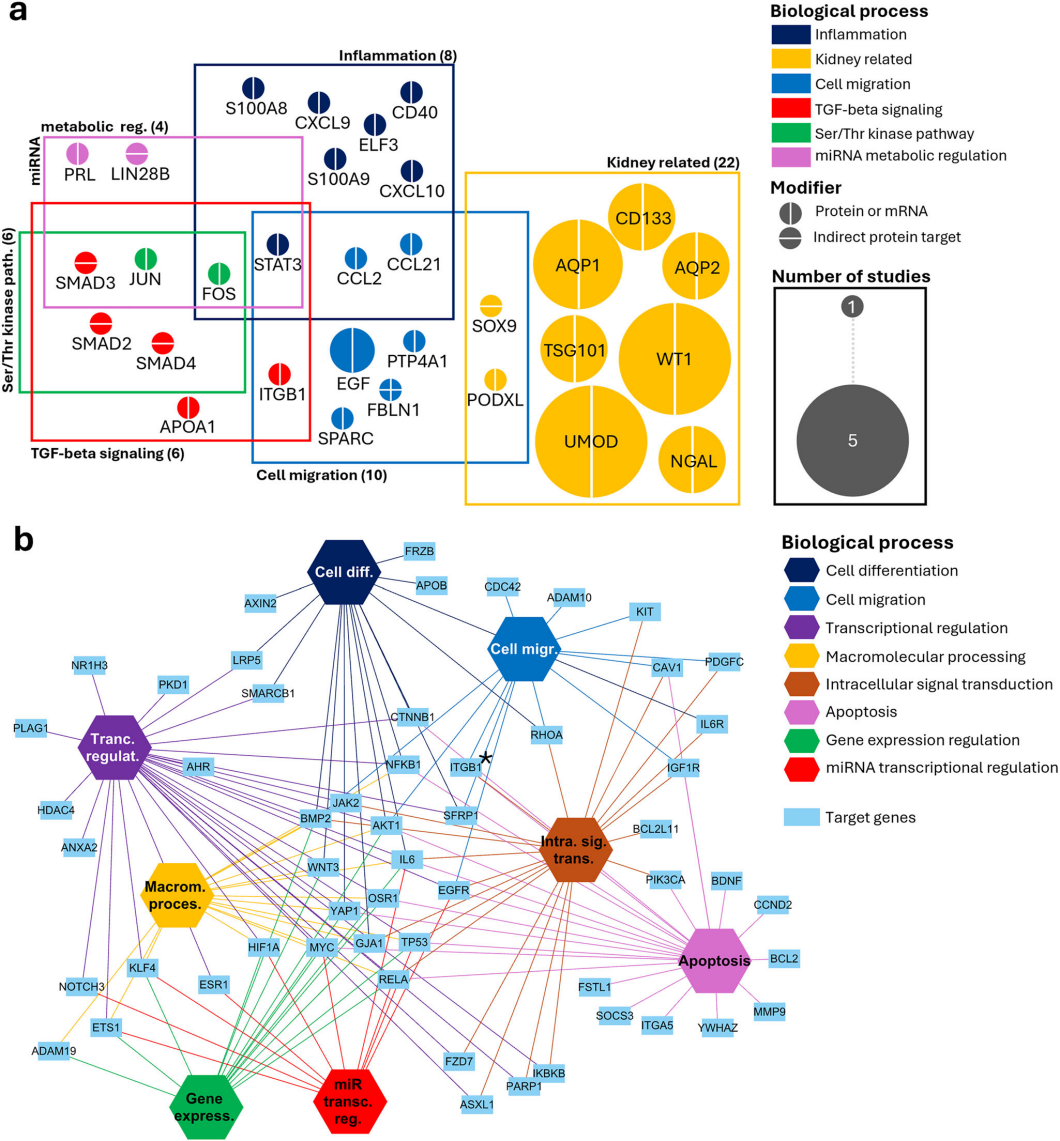
Proteins (a) and miRNA targets (b) identified in key biological processes in diagnostic studies. (a) Clusters represent biological processes associated with the identified proteins, as determined by Gene Ontology (GO) analysis. Node inner and outline colours indicate the primary and secondary key functional category of each protein related to kidney physiology, while node size reflects the frequency of its occurrence across the reviewed studies. (b) Target–pathway network illustrating the connections between miRNA target genes that catalogued and found strong evidence on miRTarBase and their associated biological processes. Target genes that were experimentally studied and are also present in panel (a) are indicated with an asterisk.

Here, we focused on the most frequently reported, altered, or proposed biomarker proteins from the reviewed studies to investigate the cellular processes in which they are potentially involved. GO term enrichment analysis for biological processes revealed major terms related to the regulation of kidney physiopathology, cell migration, TGF‐β signalling and inflammation (Figure 6a). These enriched pathways well reflect the dynamic cellular responses occurring during tubular injury and maladaptive repair with EMT and fibrotic remodelling. Additional enriched terms, including serine/threonine signal transduction pathways, and miRNA metabolic regulation, further suggest that EV‐associated proteins are involved in pathways governing cell activation and metabolism, indicating that EV cargo may reflect upstream regulatory networks involved in disease progression.

Among the repeatedly studied proteins among the diagnostic studies were kidney specific proteins: uromodulin, aquaporins, CD133, NGAL, and WT1. Uromodulin, also known as Tamm–Horsfall protein, is a glycoprotein abundantly expressed in epithelial cells of the thick ascending limb of the loop of Henle, where it regulates ion transport and water balance in the nephron. In serum, it has been reported as a predictor of tissue injury in patients with glomerulonephritis (Tachibana et al. 2019). In an observational study involving 242 participants, urinary EVs were isolated from patients with diabetic kidney disease DKD, type 2 diabetes, and CKD to investigate changes in mRNA profiles of candidate genes (Yamamoto et al. 2018). A significant increase in uromodulin mRNA levels was observed in both mild and severe DKD groups compared to healthy controls, correlating with albumin‐to‐creatinine ratio, estimated glomerular filtration rate (eGFR), and HbA1c. Uromodulin mRNA levels in urinary EVs were also higher in DKD compared to diabetes without kidney involvement. Another study proposed urinary uromodulin as an early diagnostic biomarker for diabetic nephropathy (Barr et al. 2024), reporting significantly increased uromodulin mRNA in EVs and urinary protein levels in early‐stage disease. The authors suggested that uromodulin could serve as an early diagnostic marker, potentially preceding the onset of moderately elevated albuminuria.

Aquaporins AQP1 and AQP2 are water channel proteins responsible for kidney water reabsorption and have been detected in urinary EVs. Multiple studies have reported marked reductions in AQP1 and AQP2 levels in patients with CKD, kidney allografts, and in rat models of AKI. Findings from a kidney transplantation study indicated a significant decrease in urinary EV‐derived AQP2 in recipients with high urine output and low urine osmolality on day 1 post‐transplantation, with levels progressively recovering by day 6 (Oshikawa‐Hori et al. 2019). In contrast, AQP1 levels also declined but without statistical significance. Authors proposed that reduced AQP2 expression may contribute to acute diuresis in kidney allograft recipients. Consistent with these findings, decreased AQP1 and AQP2 levels were also reported in AKI rat models developing fibrosis (Asvapromtada et al. 2018). In that study, two models of kidney ischemia reperfusion injury were used: bilateral and unilateral, and animals were monitored for 35 days. In the bilateral model, which induced mild injury, a reduction in AQP1 and AQP2 in urinary EVs was observed on day 3 but began to recover by day 7. Conversely, in the unilateral model, which caused more severe injury, a significant decrease in both aquaporins was evident on day 7 and remained low at day 35. Based on these results, authors suggested that changes in AQP levels in urinary EVs may serve as indicators for monitoring the transition from AKI to CKD.

Another important protein investigated in the context of kidney transplantation and kidney diseases is CD133, a renal progenitor marker. One study reported that urinary EVs from kidney insufficient patients lacked CD133^+^ vesicles, in contrast to healthy individuals (Dimuccio et al. 2014). Immunoblot analyses further demonstrated that sorted CD133^+^ vesicles contained glomerular and proximal tubular markers, underscoring their kidney origin. In kidney transplantation, urinary CD133 levels were found to be increased at day 7 in patients undergoing kidney damage with delayed graft function. These findings were reinforced by a longitudinal study of 58 kidney transplant recipients, which profiled 37 EV surface markers from serum and urine before transplantation and at 10–14 days, 3 months, and 1 year afterward (Burrello et al. 2023). That study revealed a progressive increase in mesenchymal progenitor cell markers (CD1c, CD105, and SSEA‐4), including CD133, in urinary vesicles of patients displaying renal recovery at follow‐up. The EV levels of progenitor markers were able to predict patient outcome at early time point analyses, suggesting that the sustained release of CD133^+^ urinary EVs may reflect nephron regenerative ability and functional recovery. Complementing these observations, another study proposed CD133 as a tool to monitor kidney physiology, showing reduced urinary CD133 expression in chronic glomerulonephritis (Dimuccio et al. 2020).

#### EV Content: RNA

Among RNA types, miRNAs are the most extensively studied, with a comparable number of publications to those investigating EV proteins (Figure 5). They have been explored across almost all disease types, either through high‐throughput profiling such as microarray analysis for biomarker discovery or by focusing on specific candidates to validate mechanistic hypotheses. Certain miRNAs have been consistently reported across different studies, suggesting their potential as robust biomarkers. For instance, increased levels of miR‐21 have been repeatedly observed in urinary and blood‐derived EVs of patients with DKD (Florijn et al. 2019, Zang et al. 2019). Functional studies further support a pathogenic role of EV‐associated miR‐21: in vivo experiments demonstrated that EVs derived from tubular epithelial cells and injected into unilateral ureteral obstruction mice aggravated kidney fibrosis, enhanced EV secretion, and activated the PTEN/Akt pathway (Zhao et al. 2021). Inhibition of miR‐21 in EVs was shown to abolish fibroblast activation and attenuate kidney fibrosis after obstruction, highlighting its therapeutic potential. Clinically, EV miR‐21 has also been linked to kidney transplantation, where miR‐21, miR‐210, and miR‐4639 levels were found to negatively correlate with eGFR in transplant recipients (Chen et al. 2020). miR‐21 has also been implicated in systemic diseases with kidney involvement. In lupus patients, increased urinary EV levels of miR‐21, miR‐150, and miR‐29c were proposed as early biomarkers of kidney fibrosis (Solé et al. 2019). Among these, miR‐150 has attracted particular attention in DKD. Multiple independent studies showed elevated urinary and circulating EV miR‐150 in DKD patients compared to healthy controls (Kim et al. 2019, Lee et al. 2020, Xie et al. 2017). Mechanistic studies demonstrated that EVs from miR‐150‐overexpressing tubular epithelial cells are directly internalized by fibroblasts, leading to their activation, proliferation, and increased profibrotic responses in ischemic mouse models (Guan et al. 2020). Conversely, inhibition of EV miR‐150 suppressed fibroblast activation *in vitro* and ameliorated fibrosis in ischemia reperfusion injury mice (Zhou et al. 2021). Together, these findings point not only to the diagnostic utility of miR‐21 and miR‐150 in urinary or circulating EVs, but also to their potential as therapeutic targets for CKD and related pathologies.

Many studies report distinct sets of EV‐derived miRNAs depending on disease context, sample source, and detection method. Although these findings may appear heterogeneous at first glance, functional enrichment analyses of their experimentally validated or predicted targets reveal convergence on common biological processes. GO enrichment analysis on kidney disease related target genes of the EV‐associated miRNAs, proposed as potential diagnostic markers, revealed that their predicted targets were predominantly involved in the regulation of cell differentiation, migration, apoptosis, transcription and miRNA regulation, and intracellular signal transduction (Figure 6b). In particular, enriched terms such as cell migration, apoptosis and transcription regulation suggest that these diagnostic marker–associated miRNAs may influence key processes of cell survival and tissue remodelling in kidney disease. Several enriched pathways were related to transcriptional regulation by RNA polymerase II, indicating a potential impact on gene expression programs that underlie the initiation and progression of fibrosis.

Based on both proteins and miRNA target genes, identified as potential diagnostic biomarkers, the enrichment analysis indicates that EV cargo is primarily associated with biological processes involved in key pathogenic events of CKD. These processes can be broadly grouped into kidney injury, fibrosis development, EMT, and cellular reprogramming. Notably, several enriched clusters highlight pathways that regulate inflammatory signalling, fibrotic remodelling, and intercellular communication within the kidney microenvironment. The presence of these pathways suggests that EV cargo may capture early molecular signalling events that contribute to CKD progression. Importantly, several of these pathways were consistently represented across different EV cargos and disease contexts, suggesting that EV‐associated molecules capture shared molecular signatures of kidney injury rather than disease‐specific events alone. Moreover, these findings support the concept that EV cargo not only mirrors tissue damage but rather reflects early events that precede structural changes, capturing the broader molecular mechanisms underlying kidney disease progression.

Other RNA species have been proposed as diagnostic biomarkers, including mRNA, circRNA, and lncRNA (Figure 5). Although they are generally considered less favourable compared to proteins and miRNAs, several strong candidates have been reported. For example, one study investigated the role of EVs derived from tubular epithelial cells in albumin‐induced tubulointerstitial inflammation (Lv et al. 2018). Analysis of EV cargo revealed that inflammatory cytokine mRNAs such as TNF‐α, IL‐6, and CCL2 were enriched both in kidney tissue and urine samples of AKI mouse models. In CKD models, CCL2 mRNA levels in EVs were also significantly increased. Functional experiments demonstrated that EVs isolated from BSA‐treated tubular epithelial cells, when transferred to macrophages, promoted an enhanced inflammatory response and macrophage migration. Moreover, administration of these EVs to albumin‐induced kidney injury models led to tubular damage and inflammatory cell infiltration. Conversely, knockdown of CCL2 in tubular epithelial cells attenuated the pro‐inflammatory effects *in vitro* and reduced kidney inflammation and tubular injury in vivo. Collectively, these findings suggest that CCL2 mRNA packaged in tubular epithelial cell‐derived EVs actively contributes to albumin‐induced tubulointerstitial inflammation by being internalized into macrophages. The same research group further examined inflammatory chemokines and cytokines in urinary EVs to identify novel diagnostic biomarkers for diabetic nephropathy (Feng et al. 2021). By isolating small urinary EVs from patients with diabetic nephropathy, diabetes mellitus, and healthy controls, they profiled a panel of inflammatory genes and found a marked upregulation of CCL21 mRNA in diabetic nephropathy patients compared to the other groups. Importantly, the increase in CCL21 mRNA in urinary EVs was detectable at early stages of diabetic nephropathy with higher accuracy than conventional clinical markers such as eGFR and albuminuria, highlighting its diagnostic potential. Moreover, CCL21 mRNA levels correlated with the severity of tubulointerstitial inflammation, and kidney biopsy analyses demonstrated that high CCL21 expression was associated with the accumulation of CD3^+^ T cells within tubular and interstitial compartments. Collectively, these findings suggest that urinary EV‐derived CCL21 mRNA represents a promising biomarker for early detection of diabetic nephropathy and also may contribute mechanistically to disease progression through T cell–mediated inflammation.

In addition to studies targeting specific RNAs, transcriptomic analyses have also been applied to EVs to uncover differentially expressed genes under disease conditions or to identify potential diagnostic biomarkers. For example, a recent study performed RNA sequencing on serum‐derived EVs from CKD patients to assess the effects of roxadustat, a therapeutic agent for anaemia in CKD (Zhou et al. 2025). The analysis identified 957 mRNAs and 914 lncRNAs that were differentially expressed following roxadustat treatment, indicating broad transcriptomic alterations in EV cargo linked to drug response. Although these findings provide insight into the molecular impact of roxadustat in CKD, further studies with larger patient cohorts and independent validation are needed to confirm the clinical utility of serum EV transcriptomics in monitoring therapeutic outcomes. Similarly, another study analysed EVs derived from high‐glucose treated HK2 cells to mimic diabetic nephropathy and reported 169 lncRNAs, 885 mRNAs, 3 circRNAs, and 152 miRNAs as differentially expressed (Zhou et al. 2021). Among these, several RNAs gained particular attention due to their potential functional or biomarker roles. For instance, EVs enriched in miR‐486 from human endothelial colony‐forming cells were shown to protect against ischemic cell death and kidney function loss, highlighting therapeutic potential (Livingston and Wei 2016); notably, miR‐486 expression was reduced in the high‐glucose group. In addition, hsa_circ_0004771, one of the identified circRNAs, has previously been proposed as a diagnostic biomarker for colorectal cancer (Pan et al. 2019), suggesting possible cross‐disease biomarker relevance. Collectively, these transcriptomic studies demonstrate how EV‐associated RNAs may serve as a resource for biomarker discovery and mechanistic insights in kidney diseases.

Some studies have focused specifically on circRNAs and lncRNAs in EVs. For example, one study investigated hsa_circ_0008925, whose host gene *SEC63* was found to correlate with kidney interstitial inflammation (Cao et al. 2022). They reported that expression of hsa_circ_0008925 was increased in EVs isolated from TGF‐β1–induced proximal tubular cells compared with controls, and this finding was further validated in urinary EVs from patients with glomerular disease. Importantly, elevated levels of this circRNA in urinary EVs correlated with clinical parameters including serum creatinine, blood urea nitrogen, eGFR, and cystatin C. The same group also performed a circRNA microarray analysis on urinary EVs from CKD patients and observed decreased levels of hsa_circ_0036649 in patients with kidney fibrosis (Cao et al. 2022). Together, these findings suggest that circRNAs carried by urinary EVs may serve as promising non‐invasive biomarkers for CKD and kidney fibrosis.

#### Other EV Contents

There are relatively few studies investigating EV components other than proteins and nucleic acids in CKD‐related diseases (Figure 5). One study focused on the presence of cAMP in urinary EVs of patients with ADPKD (Rosenberg et al. 2023). Previous reports from *in vitro* experiments, animal models, and patient biopsy analyses have demonstrated reduced intracellular calcium levels due to PKD1 or PKD2 mutations, which are associated with elevated cAMP levels in cystic tissue. In this context, authors suggested that while urinary cAMP decreased in correlation with eGFR, cAMP levels in urinary EVs showed a bimodal pattern: they initially increased during kidney enlargement and then decreased with cyst detachment from the tubular tree, accompanied by macrophage recruitment and fibroblast activation by “cystic EVs.” These processes contributed to disruption of tissue architecture and fibrosis. This observation was partially supported by another study showing higher intracellular cAMP levels in cystic mice compared to non‐cystic mice, indicating stage‐dependent alterations in ADPKD (Fonseca et al. 2014). Due to these bimodal changes in urinary EVs, cAMP does not appear to be a reliable diagnostic biomarker at present, but the findings may help clarify mechanisms of ADPKD progression. Another study examined the role of the IKK2/NF‐κB signalling pathway in vascular calcification associated with CKD (Miyazaki‐Anzai et al. 2024). The authors observed local activation of IKK2/NF‐κB in VSMCs, which coincided with increased vascular stiffness and calcification. Interestingly, pharmacological inhibition of the pathway did not mitigate these effects but instead exacerbated vascular mineralization, stiffness, and EV calcification. Previous studies have similarly implicated calcified EVs in vascular osteogenesis and mineralization (Chen et al. 2018, Kapustin et al. 2015, Rogers et al. 2020). Notably, when cell death was experimentally blocked, the researchers reported reduced VSMC mineralization and decreased calcified EV release. Collectively, these findings suggest that activation of the IKK2/NF‐κB pathway may help limit the release of calcified apoptotic bodies and thus prevent vascular stiffening, highlighting a complex and context‐dependent role of this pathway in vascular calcification.

### 3.2 EVs as Therapeutic Tools

Several studies have explored the potential therapeutic applications of EVs in CKD, given their influence on various cellular processes in target cells. These approaches include producing EVs in cell culture, isolating them from biological fluids, or modifying them to enhance their targeting or functionality. This section outlines the EV sources and cargo types that have been proposed or utilized for therapeutic purposes in CKD and related diseases.

#### EV Source: MSCs

**FIGURE 7:**
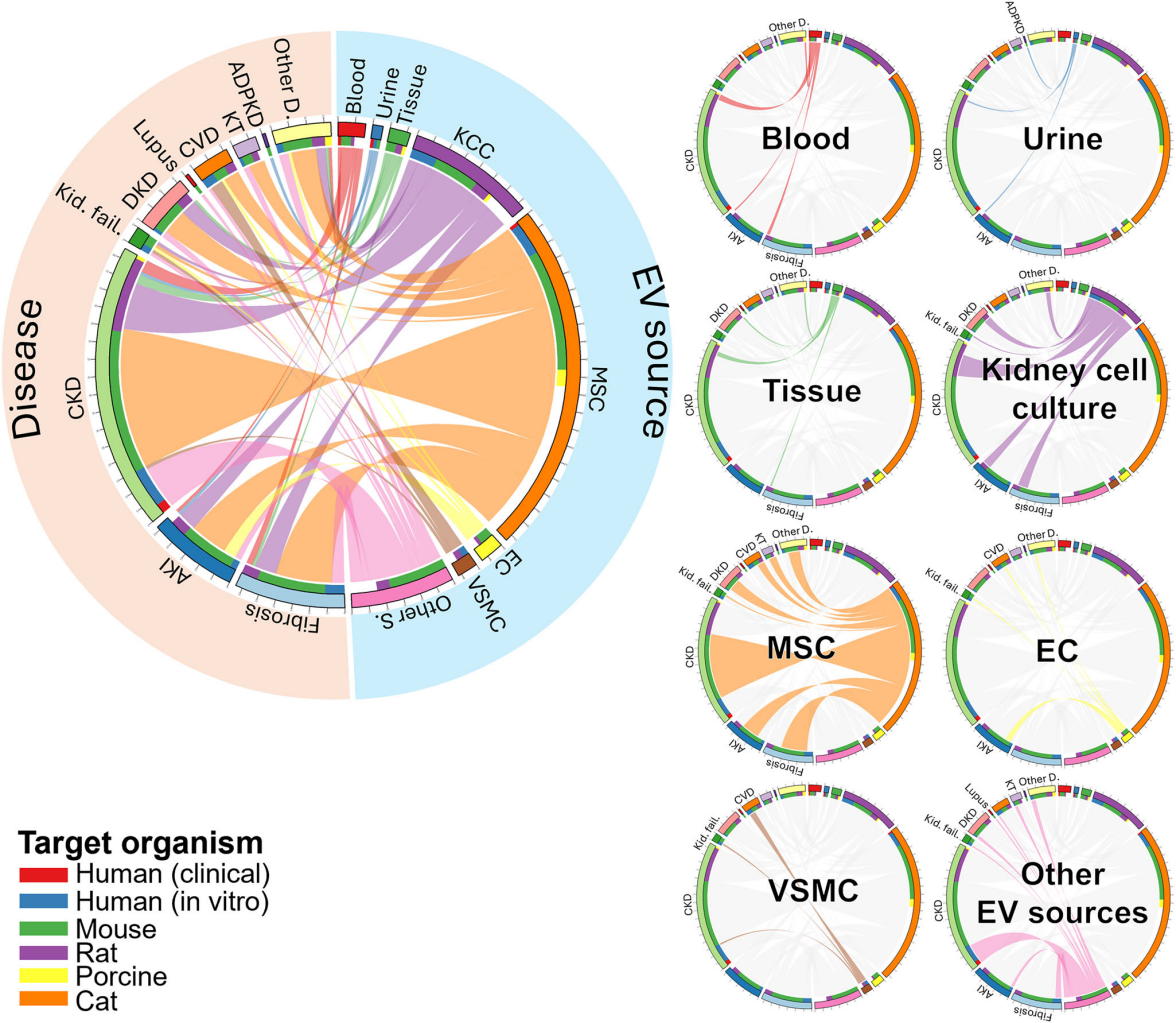
Chord diagram of therapeutic studies with EV source. The outer layer represents EV source and disease types, while the links indicate the distribution of specific EV sources across diseases. The inner layer represents the target organism used in the study (organism studied). The ticks on the outer layer indicates every 5 records. To enhance clarity of links, EV sources were also highlighted individually. KCC: kidney cell culture, MSC: mesenchymal stem cell, EC: endothelial cell, VSMC: vascular smooth muscle cell, Other S: other EV sources, AKI: acute kidney injury, CKD: chronic kidney disease, Kid. fail.: kidney failure, DKD: diabetic kidney disease, CVD: cardiovascular diseases, KT: kidney transplantation, ADPKD: autosomal dominant polycystic kidney disease, OtherD: other diseases.

Most therapeutic studies on EVs are based on cell culture sources (Figure 7). Among these, MSC‐derived EVs account for nearly half of the studies and are connected to a wide range of diseases. MSCs have long attracted interest in regenerative medicine because they can be easily isolated from various tissues, expanded *in vitro*, and differentiated into multiple cell types (Mei et al. 2024). MSC‐derived EVs are particularly appealing since they carry lower levels of MHC and other membrane‐bound proteins, making them less detectable by immune cells and representing a safer alternative to cell‐based therapies (Keshtkar et al. 2018). A recent study investigated the therapeutic effects of EVs derived from adipose‐derived MSCs (AMSCs) on kidney function (Zhang et al. 2025), building on previous evidence of their beneficial effects in wound healing (An et al. 2021) and cancer therapy (Hamilton and Teufelsbauer 2022). In a ureteral obstruction mouse model, treatment with EVs isolated from hypoxia‐stimulated AMSCs improved kidney function by reducing TNF‐α and IL‐6 levels and inhibiting apoptosis. Likewise, administration of umbilical cord‐MSC‐derived EVs in kidney transplantation injury attenuated senescence‐ and apoptosis, reduced tubular atrophy and fibrosis, and acted through regulation of the Ras‐pERK pathway (Ma et al. 2025). These findings underscore the therapeutic potential of HUMSC‐EVs in mitigating tissue injury following kidney transplantation by targeting cellular senescence and apoptosis. Importantly, in a genetic CKD model with amniotic fluid stem cell (AFSC)‐EV administration, the use of spatial transcriptomic analysis demonstrated a specific regulation in the glomerular tissue of pathways altered during disease progression and, in particular, the induction of pro‐regenerative processes (Dedhia et al. 2026). A different MSC‐EV mechanism of action might be due to the modulation of the extracellular environment, supporting the role of EVs as molecular scavengers. In particular, AFSC‐EVs were reported to carry VEGF receptors (VEGFR1 and VEGFR2), enabling them to sequester excessive VEGF, whose signalling was markedly elevated in the glomeruli of Alport mice. By trapping VEGF, EV treatment attenuated VEGF‐induced endothelial damage and preserved glomerular integrity (Sedrakyan et al. 2017).

An important series of studies has been performed in porcine models. One study investigated the effect of adipose‐derived MSC‐EVs on microvascular loss in renovascular disease (Eirin et al. 2018). In this work, swine with unilateral renovascular disease complicated by metabolic syndrome were treated with MSC‐derived EVs. After four weeks, labelled EVs were detected in tubular and endothelial cells of the stenotic kidney. EV treatment restored angiogenic factors, improved cortical microvascular and peritubular capillary density, and attenuated apoptosis, oxidative stress, and fibrosis. In a follow‐up study, the same group demonstrated that MSC‐derived EVs improved kidney function and reduced tissue injury in pigs with diet‐induced metabolic syndrome and kidney artery stenosis (Zhao et al. 2020). While both MSC and EV treatments produced similar outcomes, the authors proposed distinct mechanisms: MSCs primarily preserved microcirculation, whereas EVs maintained cellular integrity. These findings highlight the therapeutic potential of EV‐based approaches and point to mechanistic differences compared with cell‐based therapies.

In addition to cell culture and animal model studies, a clinical trial has also been conducted to evaluate the therapeutic potential of MSC‐derived EVs in patients with stage III and IV CKD. In this study, human umbilical cord‐MSC‐derived EVs were administered in two doses, which resulted in a significant increase in plasma levels of TGF‐β1, IL‐10, and TNF‐α, along with reduced mortality and improved kidney function (Nassar et al. 2016). Although clinical evidence is still limited, the therapeutic application of EVs is rapidly gaining interest, with similar approaches already explored in other conditions such as COVID‐19, ARDS, ulcers, and perianal fistula. Notably, nearly 60% of therapeutic EV studies to date have utilized MSC‐derived EVs (Mizenko et al. 2024).

#### EV Source: Kidney Cell Culture

Another commonly used EV source in therapeutic studies is KCC, which are frequently applied across different disease models (Figure 7). An important study aimed at achieving complete kidney tissue regeneration in CKD developed a bioactive scaffold composed of bioactive components, intermediate mesoderm cells, and EVs isolated during their differentiation into kidney progenitor cells (Cha et al. 2023). Application of this scaffold in a 3/4 nephrectomy mouse model promoted regeneration of the injured kidney, as indicated by reduced blood urea nitrogen and creatinine levels, together with increased glomerular size and angiogenesis. In a subsequent study, they enhanced the scaffold by incorporating multifunctional EVs engineered by SDF1α overexpression—designed to recruit stem cells to the site of injury—and further loaded with edaravone, a radical scavenger with protective effects against kidney damage (Lee et al. 2024). When tested in a 5/6 nephrectomy mouse model, this improved scaffold further promoted tubular regeneration, as reflected by increased expression of AQP1 and CDH16. In their most recent work, they developed a multiplexed scaffold containing melatonin‐modulated EVs derived from HUMSCs, which demonstrated therapeutic efficacy in severe CKD mouse models (Rhim et al. 2025). Collectively, these findings indicate that biodegradable bioactive scaffold technologies incorporating EVs hold strong potential as regenerative treatments for CKD.

Therapeutic studies using EVs derived from KCC have also been conducted in different disease models. For instance, BIRI mice treated with tubular cell‐derived EVs showed improved microvascular flow, reduced coagulation, and better kidney function and histology, the potential therapeutic role of tubular EVs in AKI (Dominguez et al. 2022). Similarly, another study tested tubular cell‐derived EVs in a rat model of DN and found attenuation of kidney injury, along with modulation of high glucose–induced miRNAs and growth factors, based on kidney histopathology (Fakhredini et al. 2022). Therapeutic applications have also been explored in systems designed to mimic kidney function. In one study, a tubule‐on‐a‐chip model was established using a hollow fibre membrane seeded with tubuloid‐derived cells in an immature state (Lindoso et al. 2022). EVs isolated from proximal tubular cells engineered with an anion transporter, OAT1, were applied to this system, leading to enhanced functional transport activity and improved maturation of tubuloid‐derived cells. Based on these findings, the authors proposed the potential use of EVs in combination with tubuloid‐derived cells to generate tight polarized epithelial monolayers for bioartificial kidney applications. Collectively, these results suggest that kidney tubular cell‐derived EVs may exert protective and regenerative effects across different forms of kidney injury, supporting their potential as a versatile therapeutic tool.

#### EV Source: Biological Fluids

Although biological fluids such as blood and urine are the most commonly used EV sources in diagnostic studies, relatively few therapeutic applications have been reported (Figure 7). A recent study isolated urinary EVs from healthy mice and identified the presence of polycystins (PC1 and PC2) (Huang et al. 2025). Indeed, the delivery of polycystins through urinary EVs to diseased mouse models of polycictic kidney disease rescued the genetic defects, resulting in reduced kidney size, lower cyst index, and increased PC1 expression. Similarly, urine‐derived EVs also resulted to display a therapeutic effect for kidney injury, due to the presence of Klotho, an anti‐aging and reno‐protective protein (Huang et al. 2025). Collectively, these findings highlight the therapeutic potential of EVs derived from biological fluids, either as carriers of functional molecules or as direct modulators of disease processes in kidney disorders.

#### EV Source: Tissues

Like biological fluids, tissues have not been widely studied as therapeutic sources of EVs. However, a recent study hypothesized that reprogramming EV secretion in skeletal muscle through metabolic engineering may provide a therapeutic approach for CKD (Zhao et al. 2025). During chronic tissue injury, which causes mitochondrial damage and loss of muscle mass, skeletal muscle‐derived EVs were shown to contribute to disease progression. By contrast, high‐intensity exercise, or adenovirus‐mediated muscle mitochondrial transcription factor A overexpression, enhanced mitochondrial activity, increased EV secretion, and altered EV cargo, including proteins involved in oxidative phosphorylation, lipid and ATP metabolism. When CKD mouse models were treated with these *in vivo* reprogrammed muscle‐derived EVs, the vesicles were detected in multiple organs such as the liver, lung, kidney, and spleen. In skeletal muscle, treatment restored mitochondrial mass and structure and preserved vascular network integrity. In the kidney, it reduced proinflammatory cell infiltration, cytokine expression, myofibroblast activation, and matrix deposition. Based on these findings, the researchers proposed that reprogrammed skeletal muscle‐derived EVs may contribute to kidney repair while simultaneously attenuating tissue injury in skeletal muscle, highlighting tissue‐derived EVs as an emerging therapeutic avenue in CKD.

#### EV Source: Endothelial Cells and Vascular Smooth Muscle Cells

Although only a limited number of studies have investigated endothelial cells and VSMCs as EV sources (Figure 7), several key findings suggest promising therapeutic outcomes in CKD‐related diseases. One research group focused on the nephroprotective effects of amniotic endothelial cells (AECs) in AKI and sepsis‐associated AKI. Proteomic analysis of AEC‐derived EVs revealed enrichment in extracellular matrix organization, growth factor signalling pathways, cytokine regulation, and immunomodulatory functions. Treatment of IRI mice with these EVs improved survival and attenuated kidney injury by modulating apoptosis, angiogenesis, and immune responses (Ren et al. 2020). In a follow‐up study demonstrated a nephroprotective role of AEC‐derived EVs against cisplatin‐induced nephrotoxicity, suggesting their potential use to mitigate the nephrotoxic side effects of chemotherapy (Kang et al. 2021). Consistent therapeutic benefits were also observed in sepsis‐associated AKI models, further supporting the protective capacity of endothelial‐derived EVs (Chi et al. 2022).

As indicated in the diagnostic chapter, VSMC‐derived EVs are key contributors to vascular calcification in CKD patients. One study demonstrated that EVs released from VSMCs share similar contents with osteoblast‐derived EVs, including calcium‐binding and extracellular matrix proteins, and that elevated calcium levels promote the secretion of calcifying EVs (Kapustin et al. 2015). Another study reported a direct interaction between VSMC‐derived EVs and type I collagen, which facilitates calcium crystallization; however, treatment of isolated rat aortic rings with DP8, a specific oligogalacturonic acid, reduced the expression of osteogenic markers and prevented the interaction between collagen I and calcified VSMC‐derived EVs (Hodroge et al. 2017). More recently, miRNA profiling identified miR‐32 as the most differentially expressed miRNA in VSMC‐derived EVs, where it was shown to promote the osteoblast‐like transition of calcified VSMCs through the PTEN/PI3K/Akt signalling pathway (Guo et al. 2025). Additionally, administration of the Bushen Huoxue formula, a traditional Chinese medicine that was shown its clinical efficacy in CKD, reduced miR‐32 levels in VSMC‐derived EVs and enhanced PTEN expression in the aortas of CKD rats with vascular calcification. Collectively, these findings highlight the pathological impact of calcified VSMC‐derived EVs on vascular calcification and suggest that targeting either their interactions with extracellular matrix components or their molecular cargo may offer a strategy to limit disease progression in CKD.

#### Other EV Sources

Several other sources, such as stem cells (adipose, dental pulp, liver, induced pluripotent, and satellite), immune cells (macrophages, natural killer, and regulatory T cells), and cardiosphere cells, have been explored in CKD‐related diseases. One notable study evaluated the use of adipose stem cell–derived EVs as an adjuvant to Losartan, an angiotensin II receptor blocker frequently used in CKD treatment (Noda et al. 2025). In rats subjected to 5/6 kidney ablation, co‐administration of Losartan and EVs significantly reduced glomerulosclerosis and interstitial infiltration while improving kidney function. Another study developed macrophage‐derived EVs loaded with Losartan to cross the blood–brain barrier and enhance drug delivery (Tan et al. 2025). In adriamycin‐induced nephropathy mice, these EVs reduced serum creatinine, blood urea nitrogen, tubular injury, and glomerulosclerosis, demonstrating antifibrotic and anti‐inflammatory effects through downregulation of the renin–angiotensin system. Together, these findings highlight EVs as promising adjuvants or delivery vehicles that enhance Losartan's therapeutic efficacy in CKD models.

#### EV Content: Protein

**FIGURE 8:**
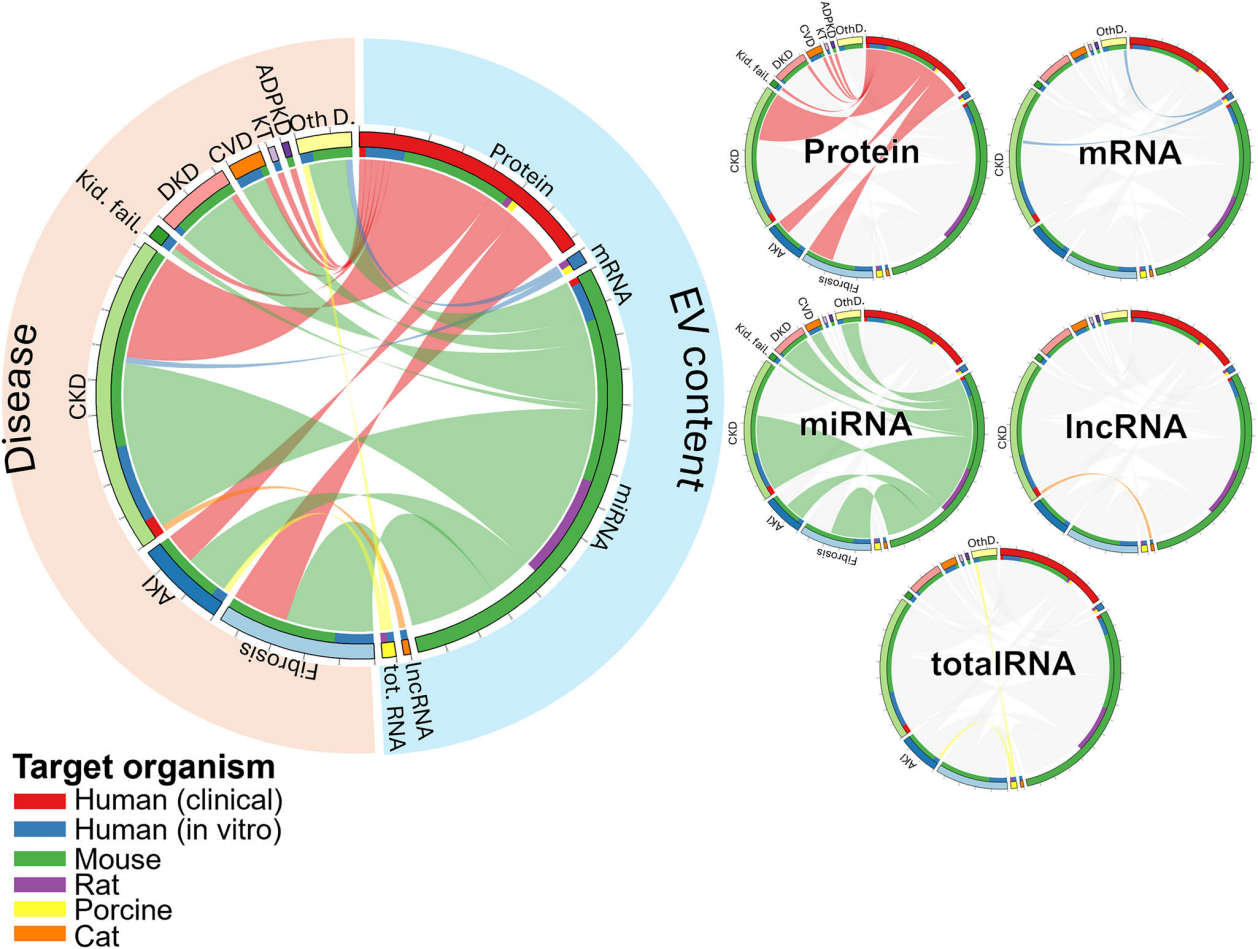
Chord diagram of therapeutic studies with EV content. The outer layer represents EV content and disease types, while the links indicate the distribution of specific EV content across diseases. The inner layer represents the target organism used in the study (organism studied). The ticks on the outer layer indicates every 5 records. To enhance clarity of links, EV contents were also highlighted individually. tot. RNA: total RNA, Other C: other EV contents, AKI: acute kidney injury, CKD: chronic kidney disease, Kid. fail.: kidney failure, DKD: diabetic kidney disease, CVD: cardiovascular diseases, KT: kidney transplantation, ADPKD: autosomal dominant polycystic kidney disease, OtherD: other diseases.

As for diagnostic studies, most therapeutic mechanisms described have been associated to EV carried proteins (Figure 8). EVs were not only used as naive, but many approaches also focused on engineering them to deliver proteins to injured tissues and to enhance their intrinsic therapeutic activity. One example is the engineering of MSC‐derived EVs for AKI treatment by overexpressing the transcription factor Oct‐4, which is known to stimulate mesenchymal‐epithelial transition of fibroblasts (Zhang et al. 2020). When hypoxic tubular epithelial cells were cultured with these engineered EVs, a significant reduction in apoptosis and an increase in proliferation were observed. Similarly, injection of engineered EVs into ischemia‐reperfusion mouse models resulted in decreased serum creatinine and blood urea nitrogen levels, along with improved kidney fibrosis. In another study, the therapeutic capacity of MSC‐derived EVs was enhanced and their half‐life prolonged by encapsulating them in an MMP2‐responsive hydrogel (Zhou et al. 2019). This design allowed a controlled release of EVs: once the hydrogel reached the injured tissue, it was degraded by a metalloprotease, MMP2, thereby releasing the EVs. Treatment of IRI mouse models with this system significantly reduced tubular cell apoptosis, proinflammatory cytokine expression, macrophage infiltration, and kidney fibrosis compared to treatment with either EVs or hydrogel alone.

In addition, several studies have explored the use of red blood cell (RBC)‐derived EVs as therapeutic carriers due to their high biocompatibility, biosafety, nonimmunogenicity, abundance, and lack of nuclear or mitochondrial DNA (Usman et al. 2018). In one such study, RBC‐derived EVs were engineered with Kim‐1–recognizing peptides to specifically target injured tubular epithelial cells and were further loaded with siRNAs against P65 and Snai1. Administration of these engineered EVs to IRI mice reduced tubulointerstitial inflammation and fibrosis, ultimately preventing CKD progression.

Alternatively, some studies have sought to modify the EV‐producing cell source to generate therapeutic EVs. For example, macrophages were genetically modified to knock out TREM‐2, a receptor implicated in kidney fibrosis (Xiao et al. 2025). EVs derived from TREM2‐/‐ macrophages exhibited an increased MMP‐9/TIMP‐1 ratio compared with EVs from wild‐type macrophages, suggesting a potential antifibrotic effect. RNA‐seq analysis further indicated that this shift in the MMP‐9/TIMP‐1 ratio was mediated through the HSPa1b/AKT pathway. Moreover, treatment of proximal tubular cells with TREM2‐/‐ macrophage‐derived EVs led to downregulation of fibrosis‐associated genes, including collagen‐1 and α‐SMA. Although the direct therapeutic effects of these EVs in vivo were not evaluated, the study demonstrated that inhibition of TREM2 in UUO mice—achieved either by injecting an adenovirus carrying shTREM‐2 or by administering a polyclonal antibody targeting TREM2—attenuated kidney fibrosis. These findings suggest that targeting TREM2 in macrophages can inhibit kidney fibrosis through, at least in part, an EV‐dependent mechanism.

**FIGURE 9:**
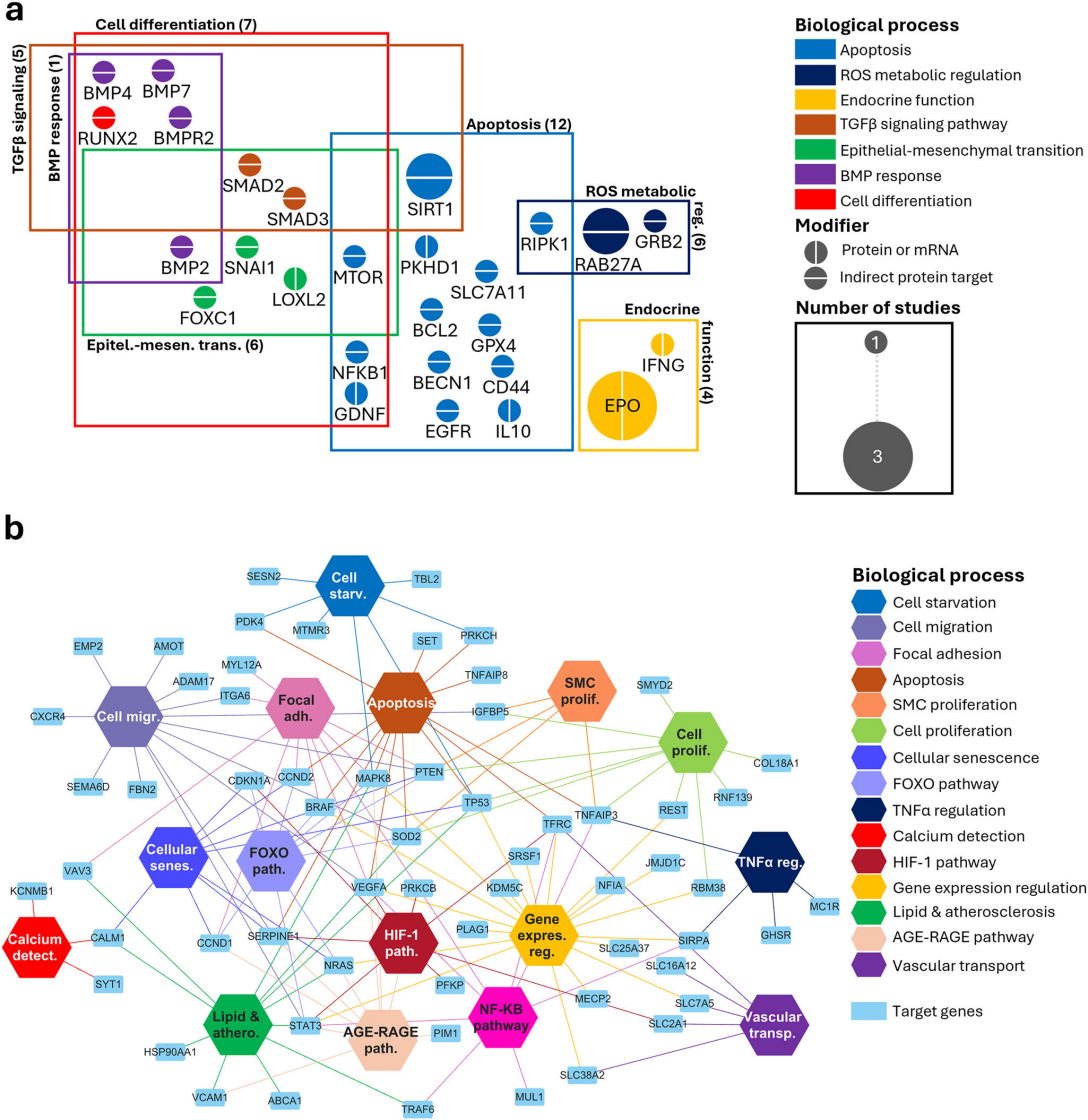
Proteins (a) and miRNAs (b) identified in key biological processes in therapeutic studies. (a) Clusters represent biological processes associated with the identified proteins, as determined by Gene Ontology (GO) analysis. Node inner and outline colors indicate the primary and secondary key functional category of each protein related to kidney physiology, while node size reflects the frequency of its occurrence across the reviewed studies. (b) Target–pathway network illustrating the connections between miRNA target genes that catalogued and found strong evidence on miRTarBase and their associated biological processes.

To better understand the molecular focus of therapeutic studies, we performed enrichment analysis of the targeted proteins using GO database. GO biological process analysis highlighted the TGF‐beta pathway and the EMT as the main enriched pathways together with the regulation of apoptosis and ROS. This analysis highlights the main therapeutic focuses on mechanisms of acute renal cell injury and transition towards fibrosis (Figure 9a).

Among the proteins identified in therapeutic studies, erythropoietin (EPO) has been repeatedly highlighted for its impact on EV biology and potential therapeutic effects in CKD. EPO is a kidney‐derived cytokine commonly used to treat anaemia in CKD, and several studies suggest that it also modulates EV content and function. For example, EVs isolated from EPO‐treated MSCs showed an altered miRNA profile and exerted anti‐apoptotic and anti‐fibrotic effects on proximal tubular cells *in vitro*, while administration of these EVs to UUO mice reduced fibrotic markers, serum creatinine, and blood urea nitrogen (Wang et al. 2015). Importantly, although untreated MSC‐derived EVs also improved outcomes, EPO stimulation amplified these beneficial effects, increasing the overall amount of secreted EVs and leading to upregulation of nearly 70% of their miRNA cargo. Consistently, another study demonstrated that EPO treatment in UUO mice increased circulating EV levels and enhanced antifibrotic activity, partly through upregulation of miR‐144, which targets tPA activation in kidney fibroblasts (Zhou et al. 2016). Furthermore, genetically engineered kidney MSCs were designed to secrete EPO‐loaded EVs, which, when administered to CKD mice with anaemia, led to increased haemoglobin, improved kidney function, and reduced serum creatinine after two weeks of treatment (Choi et al. 2022). Taken together, these studies suggest that EPO not only regulates EV biogenesis and cargo composition but can also enhance their therapeutic efficacy in kidney fibrosis, dysfunction, and CKD‐associated anaemia.

#### EV Content: RNA

Similar to diagnostic studies, more than half of the therapeutic reports focused on RNA as EV therapeutic content. Among these, miRNAs were the most frequently investigated, followed by mRNAs, lncRNAs, and total RNA screening. Most miRNA‐based studies employed mouse or rat models, as well as *in vitro* systems. Although one report initially appeared to be a clinical study—since it isolated urinary EVs from human CKD patients for biomarker discovery—it also demonstrated that targeting specific miRNAs attenuated kidney injury *in vitro* and in mouse CKD models (Kumari et al. 2020).

Among miRNA studies, some of them have shown promising therapeutic outcomes. One of these studies targeted let‐7i‐5p in kidney tubular epithelial cells by delivering its antagomir (anti‐let‐7i‐5p) with MSC‐derived EVs (Jin et al. 2021). Let‐7i‐5p was found to involve in cell proliferation and migration and targets TSC1, negative regulator of mTOR signalling. Overexpression of TSC1 decreased kidney fibrosis in diabetic nephropathy by inactivating mTOR signalling. When they treated UUO mice with MSC‐derived EVs engineered with anti‐let‐7i‐5p, reduction in kidney fibrosis including attenuated extracellular matrix deposition and EMT and improved kidney function with increased TSC1 gene expression were observed. Another study worth noting investigated the role of miR‐146a, a regulator of inflammatory response and immune balance through the NF‐κB pathway, in CKD and its modulation by Niaodukang mixture (NDK), a traditional Chinese medicine known to lower serum creatinine levels (Liu et al. 2024). In CKD rat models, miR‐146a expression was reduced in ileum‐derived EVs but increased in serum‐derived EVs, while NDK treatment normalized these alterations, suppressed microinflammation, and regulated EV secretion. These findings suggest that the therapeutic effect of NDK in CKD may, at least in part, involve the prevention of ileum‐derived EV release carrying miR‐146a, while also underscoring the influence of EV fluctuations on disease pathology.

miRNAs have also been employed to load EVs for therapeutic purposes. A study has modified MSC‐derived EVs with targeting peptides to enhance delivery efficiency and achieve optimal biodistribution within fibrotic kidney tissue (Zhang et al. 2025). Treatment with these engineered EVs in UUO mice promoted their accumulation in hypoxic endothelial cells and injured kidney tissue, leading to increased endothelial cell viability and restoration of peritubular capillaries. The therapeutic benefits of modified MSC‐derived EVs on peritubular capillary loss and kidney injury were hypothesized to be mediated, at least in part, by miR‐126‐5p, one of the predominant miRNAs carried by MSC‐derived EVs. Mechanistically, miR‐126‐5p acts as a silencer of ALKBH5, a regulatory enzyme that modulates SIRT1 expression through m6A RNA methylation. Collectively, these findings suggest that strategies to enhance the efficacy and tissue specificity of EV‐based therapies may provide improved therapeutic outcomes in kidney diseases.

Although the majority of RNA‐based therapeutic studies focused on miRNAs, a few also investigated lncRNAs, mRNAs, and total RNA sequencing. For instance, one study examined the therapeutic potential of cardiosphere cell–derived EVs after myocardial infarction (Cambier et al. 2018). Previous studies had already demonstrated that cardiosphere‐derived cells exert antiapoptotic, antifibrotic, and angiogenic effects, thereby improving tissue repair in myocardial infarction and muscular dystrophy. In hypertensive mice, injection of cardiosphere cell–derived EVs attenuated cardiac and kidney inflammation and fibrosis while improving overall function. Transcriptomic analysis revealed that the most abundant small RNA carried by these EVs was the Y RNA fragment EV‐YF1. Direct administration of EV‐YF1 reproduced similar protective outcomes, suggesting that the therapeutic effects of cardiosphere cell–derived EVs on cardiovascular and kidney injury may be mediated through EV‐YF1. In another study, MSC‐derived EVs were engineered to carry lncRNA‐Ancr, which had previously been identified as an inhibitor of osteoblast differentiation (Yan et al. 2023). Treatment of Gli1+ vascular progenitor cells with these engineered EVs promoted smooth muscle cell reconstruction while suppressing their differentiation into osteoblast‐like cells, thereby preventing vascular calcification.

To explore the biological roles of therapeutic miRNAs, we collected and analysed their predicted targets. The enrichment analysis of GO biological process terms revealed involvement in the most common physiopathological cellular processes, such as proliferation, migration, adhesion, apoptosis, and senescence (Figure 9b), highlighting a broad therapeutic effect counteracting cell damage and preserving cell integrity. In addition, the analysis also identified that intracellular pathways linked to cell survival, metabolism, and stress responses (NF‐kB, HIF‐1, TNF and FOXO pathway) were targeted. These pathways correlate with the effects of many EV‐based interventions, shown to protect tubular epithelial cells, restore microvascular integrity and endothelial function, limiting the response to injury and promoting regenerative responses within the kidney microenvironment. Interestingly, processes specifically linked to vascular calcification were identified, highlighting an additional therapeutic target within the tissue microenvironment.

Because the same enrichment analysis pipeline and thresholds were applied to both diagnostic and therapeutic datasets (Figures 6 and 9), their resulting biological processes can be directly compared within a consistent analytical framework. Reflecting their differential approach, diagnostic EV analyses appear predominantly enriched in kidney‐specific markers and pathways, mirroring localized tissue‐derived signals, whereas therapeutic EV studies target broader biological processes involving the entire kidney microenvironment, including endothelial, inflammatory, and stromal compartments. Despite this difference in scope, the biological pathways identified across diagnostic and therapeutic EV studies show substantial overlap. Diagnostic studies primarily identify EV cargoes associated with key pathogenic processes such as inflammation, apoptosis, and fibrotic signalling, while therapeutic approaches aim to modulate these same pathways to attenuate disease progression. This convergence indicates that EV‐based diagnostics and therapeutics represent complementary aspects of a shared biological framework, in which EV cargo molecules identified as biomarkers may also serve as therapeutic targets or mediators of intervention. Conversely, understanding the pathways targeted by therapeutic EVs may help refine biomarker discovery by highlighting the most biologically relevant signalling networks. Taken together, these findings support the concept that EV research in CKD should shift from descriptive biomarker discovery toward a more integrated mechanistic framework, where diagnostic and therapeutic applications are linked through shared molecular pathways.

## 4 Conclusion

In conclusion, this systematic review provides a comprehensive and structured analysis of studies investigating EVs in CKD and CKD‐related diseases, categorizing them according to EV source, molecular cargo, organism type, and disease context. Importantly, beyond their individual diagnostic or therapeutic applications, the comparative analysis of EV cargo and targets across studies reveals convergent molecular mechanisms. Functional enrichment analyses consistently highlight shared biological pathways—including TGF‐β and BMP signalling, inflammation, apoptosis, oxidative stress, and epithelial–mesenchymal transition—that underpin both EV biomarker profiles and therapeutic responses. This convergence supports the concept that EV‐based diagnostics and therapeutics are mechanistically interconnected, reflecting and targeting the same core pathogenic processes. Recognizing these shared pathways provides a unifying framework to interpret heterogeneous findings and supports the rational design of next‐generation EV‐based interventions in CKD.

With respect to clinical translation, mesenchymal stem cell–derived EVs represent one of the most promising sources due to their well‐established immunomodulatory and regenerative properties. Emerging strategies, including engineered and hybrid EVs, further enhance therapeutic potential by improving targeting capacity, cargo specificity, and functional efficacy. However, important challenges remain, including limited mechanistic understanding, and the intrinsic heterogeneity of EV populations, all of which currently constrain clinical translation. In this context, simpler vesicle systems, such as RBC‐EVs, may offer advantages due to their low nucleic acid content, reduced immunogenicity, and suitability for scalable manufacturing and clinical‐grade production.

Looking ahead, key priorities include the harmonization of EV isolation, quantification, and characterization protocols to ensure reproducibility and enable inter‐study comparison. Beyond technical standardization, a critical need is the transition from descriptive studies toward mechanism‐driven research, including rigorous definition of EV modes of action and the implementation of well‐controlled, adequately powered clinical studies. In addition, longitudinal and integrative studies combining EV multi‐omics data with imaging, histopathology, and clinical outcomes will be essential to establish causal links and assess prognostic value.

Importantly, the EV field is currently undergoing a phase of recalibration, in which initial expectations are being reassessed in light of translational and regulatory challenges. Moving beyond this phase will require prioritizing scientific rigor over hype, improving experimental design, and aligning academic discoveries with clinical and manufacturing requirements (Zarovni and Glebov 2026). In this context, the development of clearly defined diagnostic and therapeutic applications with demonstrable reproducibility, efficacy, and scalability will be essential to bridge the gap between experimental promise and clinical implementation.

## Author Contributions

**Tunahan Ergunay**: conceptualization, data curation, formal analysis, methodology, visualization, writing. **Alessia Brossa**: writing, supervision. **Benedetta Bussolati**: conceptualization, data curation, writing, supervision.

## Funding Statement

This work was supported by CN3 National Center for Gene Therapy and Drugs based on RNA Technology—Spoke 8.

## Conflicts of Interest

Authors declare no conflict of interest.

## Data Availability

The data in this study are available from the corresponding author upon reasonable request.

## References

[jev270300-bib-0001] An, Y. , S. Lin , X. Tan , et al. 2021. “Exosomes From Adipose‐Derived Stem Cells and Application to Skin Wound Healing.” Cell Proliferation 54, no. 3: e12993. 10.1111/cpr.12993. 33458899 PMC7941238

[jev270300-bib-0002] Asvapromtada, S. , H. Sonoda , M. Kinouchi , et al. 2018. “Characterization of Urinary Exosomal Release of Aquaporin‐1 and ‐2 After Renal Ischemia‐Reperfusion in Rats.” American Journal of Physiology. Renal Physiology 314, no. 4: F584–F601. 10.1152/ajprenal.00184.2017. 29357442

[jev270300-bib-0103] Behrens, F. , J. Holle , C. Y. Chen , et al. 2025. “Circulating Extracellular Vesicles as Putative Mediators of Cardiovascular Disease in Paediatric Chronic Kidney Disease.” Journal of Extracellular Vesicles 14, no. 3: e70062. 10.1002/jev2.70062. 40116365 PMC11926757

[jev270300-bib-0003] Barr, S. I. , S. S. Bessa , and T. M. Mohamed . 2024. “Abd El‐Azeem EM. Exosomal UMOD Gene Expression and Urinary Uromodulin Level as Early Noninvasive Diagnostic Biomarkers for Diabetic Nephropathy in Type 2 Diabetic Patients.” Diabetology International 15, no. 3: 389–399. 10.1007/s13340-023-00686-2. 39101162 PMC11291796

[jev270300-bib-0004] Barutta, F. , S. Bellini , S. Guarrera , et al. 2022. “Association of Serum MicroRNA‐145‐5p Levels With Microvascular Complications of Type 1 Diabetes: The EURODIAB Prospective Complications Study.” Diabetes Research and Clinical Practice 190: 109987. 10.1016/j.diabres.2022.109987. 35820565

[jev270300-bib-0005] Bikbov, B. , C. A. Purcell , A. S. Levey , et al. 2020. “Global, Regional, and National Burden of Chronic Kidney Disease, 1990–2017: A Systematic Analysis for the Global Burden of Disease Study 2017.” Lancet 395, no. 10225: 709–733. 10.1016/S0140-6736(20)30045-3. 32061315 PMC7049905

[jev270300-bib-0006] Burrello, J. , S. Monticone , A. Burrello , et al. 2023. “Identification of a Serum and Urine Extracellular Vesicle Signature Predicting Renal Outcome After Kidney Transplant.” Nephrology, Dialysis, Transplantation 38, no. 3: 764–777. 10.1093/ndt/gfac259. PMC9976747 36073758

[jev270300-bib-0007] Cambier, L. , J. F. Giani , W. Liu , et al. 2018. “Angiotensin II‐Induced End‐Organ Damage in Mice Is Attenuated by Human Exosomes and by an Exosomal Y RNA Fragment.” Hypertension 72, no. 2: 370–380. 10.1161/HYPERTENSIONAHA.118.11239. 29866742 PMC6043385

[jev270300-bib-0008] Cao, Y. , Y. Shi , Y. Wang , et al. 2022. “Exosomal hsa_circ_0008925 From Urine Is Related to Chronic Renal Fibrosis.” Disease Markers 2022: 1899282. 10.1155/2022/1899282. 35222740 PMC8881153

[jev270300-bib-0009] Cao, Y. , Y. Shi , Y. Yang , et al. 2022. “Urinary Exosomes Derived circRNAs as Biomarkers for Chronic Renal Fibrosis.” Annals of Medicine 54, no. 1: 1966–1976. 10.1080/07853890.2022.2098374. 35819256 PMC9291679

[jev270300-bib-0010] Carmona, A. , M. L. Agüera , C. Luna‐Ruiz , et al. 2017. “Markers of Endothelial Damage in Patients With Chronic Kidney Disease on Hemodialysis.” American Journal of Physiology. Renal Physiology 312, no. 4: F673–F681. 10.1152/ajprenal.00013.2016. 28077371

[jev270300-bib-0011] Carreras‐Planella, L. , J. Soler‐Majoral , C. Rubio‐Esteve , et al. 2017. “Characterization and Proteomic Profile of Extracellular Vesicles From Peritoneal Dialysis Efflux.” PLoS ONE 12, no. 5: e0176987. 10.1371/journal.pone.0176987. 28489901 PMC5425196

[jev270300-bib-0012] Carreras‐Planella, L. , J. Soler‐Majoral , C. Rubio‐Esteve , et al. 2019. “Proteomic Profiling of Peritoneal Dialysis Effluent‐Derived Extracellular Vesicles: A Longitudinal Study.” Journal of Nephrology 32, no. 6: 1021–1031. 10.1007/s40620-019-00658-3. 31617158

[jev270300-bib-0013] Cha, S. G. , W. K. Rhim , J. Y. Kim , et al. 2023. “Kidney Tissue Regeneration Using Bioactive Scaffolds Incorporated With Differentiating Extracellular Vesicles and Intermediate Mesoderm Cells.” Biomaterials Research 27, no. 1: 126. 10.1186/s40824-023-00471-x. 38049879 PMC10696796

[jev270300-bib-0014] Chen, N. X. , K. D. O'Neill , and S. M Moe . 2018. “Matrix Vesicles Induce Calcification of Recipient Vascular Smooth Muscle Cells Through Multiple Signaling Pathways.” Kidney International 93, no. 2: 343–354. 10.1016/j.kint.2017.07.019. 29032812 PMC8211355

[jev270300-bib-0015] Chen, Y. , X. Han , Y. Sun , X. He , and D. Xue . 2020. “A Circulating Exosomal MicroRNA Panel as a Novel Biomarker for Monitoring Post‐Transplant Renal Graft Function.” Journal of Cellular and Molecular Medicine 24, no. 20: 12154–12163. 10.1111/jcmm.15861. 32918330 PMC7579686

[jev270300-bib-0016] Chi, D. , Y. Chen , C. Xiang , et al. 2022. “Human Amnion Epithelial Cells and Their Derived Exosomes Alleviate Sepsis‐Associated Acute Kidney Injury via Mitigating Endothelial Dysfunction.” Frontiers in Medicine 9: 829606. 10.3389/fmed.2022.829606. 35402422 PMC8989462

[jev270300-bib-0017] Choi, H. Y. , T. Y. Kim , M. Lee , et al. 2022. “Kidney Mesenchymal Stem Cell‐Derived Extracellular Vesicles Engineered to Express Erythropoietin Improve Renal Anemia in Mice With Chronic Kidney Disease.” Stem Cell Reviews and Reports 18, no. 3: 980–992. 10.1007/s12015-021-10141-x. 33651336

[jev270300-bib-0018] Dedhia, C. , V. Villani , X. Hou , et al. 2026. “Extracellular Vesicle miR‐93‐5p Cargo Regulates Glomerular Endothelial Cell Damage in Alport Syndrome.” JCI Insight 11, no. 6: e197643. 10.1172/jci.insight.197643. 41869725 PMC13043101

[jev270300-bib-0019] Dimuccio, V. , L. Bellucci , M. Genta , et al. 2022. “Upregulation of miR145 and miR126 in EVs From Renal Cells Undergoing EMT and Urine of Diabetic Nephropathy Patients.” International Journal of Molecular Sciences 23, no. 20: 12098. 10.3390/ijms232012098. 36292960 PMC9603196

[jev270300-bib-0020] Dimuccio, V. , L. Peruzzi , M. F. Brizzi , et al. 2020. “Acute and Chronic Glomerular Damage Is Associated With Reduced CD133 Expression in Urinary Extracellular Vesicles.” American Journal of Physiology. Renal Physiology 318, no. 2: F486–F495. 10.1152/ajprenal.00404.2019. 31869243

[jev270300-bib-0021] Dimuccio, V. , A. Ranghino , L. Praticò Barbato , et al. 2014. “Urinary CD133+ Extracellular Vesicles Are Decreased in Kidney Transplanted Patients With Slow Graft Function and Vascular Damage.” PLoS ONE 9, no. 8: e104490. 10.1371/journal.pone.0104490. 25100147 PMC4123993

[jev270300-bib-0022] Dominguez, J. H. , D. Xie , J. M. Dominguez 2nd , and K. J. Kelly . 2022. “Role of Coagulation in Persistent Renal Ischemia Following Reperfusion in an Animal Model.” American Journal of Physiology. Renal Physiology 323, no. 5: F590–F601. 10.1152/ajprenal.00162.2022. 36007891 PMC9602917

[jev270300-bib-0023] Eirin, A. , X. Y. Zhu , S. Jonnada , A. Lerman , A. J. van Wijnen , and L. LO . 2018. “Mesenchymal Stem Cell‐Derived Extracellular Vesicles Improve the Renal Microvasculature in Metabolic Renovascular Disease in Swine.” Cell Transplantation 27, no. 7: 1080–1095. 10.1177/0963689718780942. 29954220 PMC6158551

[jev270300-bib-0024] Erdbrügger, U. , C. J. Blijdorp , I. V. Bijnsdorp , et al. 2021. “Urinary Extracellular Vesicles: A Position Paper by the Urine Task Force of the International Society for Extracellular Vesicles.” Journal of Extracellular Vesicles 10, no. 7: e12093. 10.1002/jev2.12093. 34035881 PMC8138533

[jev270300-bib-0025] Fakhredini, F. , E. Mansouri , S. A. Mard , A. Valizadeh Gorji , M. Rashno , and M. Orazizadeh . 2022. “Effects of Exosomes Derived From Kidney Tubular Cells on Diabetic Nephropathy in Rats.” Cell Journal 24, no. 1: 28–35. 10.22074/cellj.2022.7591. 35182062 PMC8876258

[jev270300-bib-0026] Feng, Y. , X. Zhong , H. F. Ni , et al. 2021. “Urinary Small Extracellular Vesicles Derived CCL21 mRNA as Biomarker Linked With Pathogenesis for Diabetic Nephropathy.” Journal of translational medicine 19, no. 1: 355. 10.1186/s12967-021-03030-x. 34404433 PMC8371892

[jev270300-bib-0027] Firsov, D. , and O. Bonny . 2018. “Circadian Rhythms and the Kidney.” Nature Reviews Nephrology 14, no. 10: 626–635. 10.1038/s41581-018-0048-9. 30143787

[jev270300-bib-0028] Florijn, B. W. , J. Duijs , J. H. Levels , et al. 2019. “Diabetic Nephropathy Alters the Distribution of Circulating Angiogenic MicroRNAs Among Extracellular Vesicles, HDL, and Ago‐2.” Diabetes 68, no. 12: 2287–2300. 10.2337/db18-1360. 31506344

[jev270300-bib-0029] Fonseca, J. M. , A. P. Bastos , A. G. Amaral , et al. 2014. “Renal Cyst Growth Is the Main Determinant for Hypertension and Concentrating Deficit in *Pkd1*‐Deficient Mice.” Kidney International 85, no. 5: 1137–1150. 10.1038/ki.2013.501. 24429399 PMC4510986

[jev270300-bib-0030] Fu, R. , K. Meng , R. Zhang , X. Du , and J. Jiao . 2023. “Bone Marrow‐Derived Exosomes Promote Inflammation and Osteoclast Differentiation in High‐Turnover Renal Osteodystrophy.” Renal Failure 45, no. 2: 2264396. 10.1080/0886022X.2023.2264396. 37870853 PMC11001343

[jev270300-bib-0031] Guan, H. , R. Peng , L. Mao , F. Fang , B. Xu , and M. Chen . 2020. “Injured Tubular Epithelial Cells Activate Fibroblasts to Promote Kidney Fibrosis Through miR‐150‐Containing Exosomes.” Experimental Cell Research 392, no. 2: 112007. 10.1016/j.yexcr.2020.112007. 32315664

[jev270300-bib-0032] Guo, X. , S. Liu , X. Wu , et al. 2025. “Alleviating Vascular Calcification With Bushen Huoxue Formula in Rats With Chronic Kidney Disease by Inhibiting the PTEN/PI3K/AKT Signaling Pathway Through Exosomal MicroRNA‐32.” Journal of Pharmacy and Pharmacology 77, no. 4: 550–563. 10.1093/jpp/rgae120. 39440885

[jev270300-bib-0033] Hamilton, G. , and M. Teufelsbauer . 2022. “Adipose‐Derived Stromal/Stem Cells and Extracellular Vesicles for Cancer Therapy.” Expert Opinion on Biological Therapy 22, no. 1: 67–78. 10.1080/14712598.2021.1954156. 34236014

[jev270300-bib-0034] Herrmann, I. K. , M. J. A. Wood , and G. Fuhrmann . 2021. “Extracellular Vesicles as a Next‐Generation Drug Delivery Platform.” Nature Nanotechnology 16, no. 7: 748–759. 10.1038/s41565-021-00931-2. 34211166

[jev270300-bib-0035] Hodroge, A. , E. Trécherel , M. Cornu , et al. 2017. “Oligogalacturonic Acid Inhibits Vascular Calcification by Two Mechanisms: Inhibition of Vascular Smooth Muscle Cell Osteogenic Conversion and Interaction With Collagen.” Arteriosclerosis, Thrombosis, and Vascular Biology 37, no. 7: 1391–1401. 10.1161/ATVBAHA.117.309513. 28522698

[jev270300-bib-0036] Huang, Y. , A. Osouli , H. Li , et al. 2025. “Therapeutic Potential of Urinary Extracellular Vesicles in Delivering Functional Proteins and Modulating Gene Expression for Genetic Kidney Disease.” Biomaterials 321: 123296. 10.1016/j.biomaterials.2025.123296. 40158444 PMC12048220

[jev270300-bib-0037] Jin, J. , F. Qian , D. Zheng , W. He , J. Gong , and Q. He . 2021. “Mesenchymal Stem Cells Attenuate Renal Fibrosis via Exosomes‐Mediated Delivery of MicroRNA Let‐7i‐5p Antagomir.” International Journal of Nanomedicine 16: 3565–3578. 10.2147/IJN.S299969. 34079249 PMC8164705

[jev270300-bib-0038] Kang, X. , Y. Chen , X. Xin , et al. 2021. “Human Amniotic Epithelial Cells and Their Derived Exosomes Protect Against Cisplatin‐Induced Acute Kidney Injury Without Compromising Its Antitumor Activity in Mice.” Frontiers in Cell and Developmental Biology 9: 752053. 10.3389/fcell.2021.752053. 35186944 PMC8851426

[jev270300-bib-0039] Kapustin, A. N. , M. L. Chatrou , I. Drozdov , et al. 2015. “Vascular Smooth Muscle Cell Calcification Is Mediated by Regulated Exosome Secretion.” Circulation Research 116, no. 8: 1312–1323. 10.1161/CIRCRESAHA.116.305012. 25711438

[jev270300-bib-0040] Keshtkar, S. , N. Azarpira , and M. H. Ghahremani . 2018. “Mesenchymal Stem Cell‐Derived Extracellular Vesicles: Novel Frontiers in Regenerative Medicine.” Stem Cell Research & Therapy 9, no. 1: 63. 10.1186/s13287-018-0791-7. 29523213 PMC5845209

[jev270300-bib-0041] Kim, H. , Y. U. Bae , J. S. Jeon , et al. 2019. “The Circulating sExosomal MicroRNAs Related to Albuminuria in Patients With Diabetic Nephropathy.” Journal of translational medicine 17, no. 1: 236. 10.1186/s12967-019-1983-3. 31331349 PMC6647278

[jev270300-bib-0042] Kumari, M. , A. Mohan , C. M. Ecelbarger , A. Gupta , N. Prasad , and S. Tiwari . 2020. “miR‐451 Loaded Exosomes Are Released by the Renal Cells in Response to Injury and Associated With Reduced Kidney Function in Human.” Frontiers in Physiology 11: 234. 10.3389/fphys.2020.00234. 32322216 PMC7158952

[jev270300-bib-0043] Lee, S. Y. , J. M. Park , W. K. Rhim , et al. 2024. “Multifunctional Extracellular Vesicles and Edaravone‐Loaded Scaffolds for Kidney Tissue Regeneration by Activating GDNF/RET Pathway.” Nano Convergence 11, no. 1: 43. 10.1186/s40580-024-00450-5. 39460807 PMC11512987

[jev270300-bib-0044] Lee, W. C. , L. C. Li , H. Y. Ng , et al. 2020. “Urinary Exosomal MicroRNA Signatures in Nephrotic, Biopsy‐Proven Diabetic Nephropathy.” Journal of Clinical Medicine 9, no. 4: 1220. 10.3390/jcm9041220. 32340338 PMC7231152

[jev270300-bib-0045] Li, X. , and B. Lindholm . 2023. “Cardiovascular Risk Prediction in Chronic Kidney Disease.” American Journal of Nephrology 53, no. 10: 730–739. 10.1159/000528560. 36481730

[jev270300-bib-0046] Li, X. , N. Raisinghani , A. Gallinat , et al. 2026. “Circulating Extracellular Vesicles in the Pathogenesis of Heart Failure in Patients With Chronic Kidney Disease.” Circulation 153, no. 2: 94–114. 10.1161/CIRCULATIONAHA.125.075579. 41178538 PMC12767446

[jev270300-bib-0047] Lindoso, R. S. , F. A. Yousef Yengej , F. Voellmy , et al. 2022. “Differentiated Kidney Tubular Cell‐Derived Extracellular Vesicles Enhance Maturation of Tubuloids.” Journal of Nanobiotechnology 20, no. 1: 326. 10.1186/s12951-022-01506-6. 35841001 PMC9284832

[jev270300-bib-0048] Liu, L. , L. Wang , H. Wang , et al. 2024. “Niaodukang Mixture Inhibits Micro‐Inflammation in CKD Rats by Enhancing MiR‐146a Levels in Enterogenous Exosomes.” Journal of Ethnopharmacology 332: 118318. 10.1016/j.jep.2024.118318. 38754642

[jev270300-bib-0049] Livingston, M. J. , and Q. Wei . 2016. “MicroRNAs in Extracellular Vesicles Protect Kidney From Ischemic Injury: From Endothelial to Tubular Epithelial.” Kidney International 90, no. 6: 1150–1152. 10.1016/j.kint.2016.08.032. 27884305

[jev270300-bib-0050] Lu, Y. , R. Zhang , X. Gu , X. Wang , P. Xi , and X. Chen . 2023. “Exosomes From Tubular Epithelial Cells Undergoing Epithelial‐to‐Mesenchymal Transition Promote Renal Fibrosis by M1 Macrophage Activation.” FASEB BioAdvances 5, no. 3: 101–113. 10.1096/fba.2022-00080. 36876297 PMC9983075

[jev270300-bib-0051] Lv, L. L. , Y. Feng , Y. Wen , et al. 2018. “Exosomal CCL2 From Tubular Epithelial Cells Is Critical for Albumin‐Induced Tubulointerstitial Inflammation.” Journal of the American Society of Nephrology 29, no. 3: 919–935. 10.1681/ASN.2017050523. 29295871 PMC5827595

[jev270300-bib-0052] Ma, M. , J. Zeng , M. Zhu , et al. 2025. “Human Umbilical Cord Mesenchymal Stem Cells‐Derived Extracellular Vesicles Ameliorate Kidney Ischemia‐Reperfusion Injury by Suppression of Senescent Tubular Epithelial Cells: Experimental Study.” International Journal of Surgery (London, England) 111, no. 1: 394–410. 10.1097/JS9.0000000000002074. 39236098 PMC11745712

[jev270300-bib-0053] Manoni, F. , G. Gessoni , M. G. Alessio , et al. 2012. “Mid‐Stream vs. First‐Voided Urine Collection by Using Automated Analyzers for Particle Examination in Healthy Subjects: An Italian Multicenter Study.” Clinical Chemistry and Laboratory Medicine 50, no. 4: 679–684. 10.1515/cclm.2011.823. 22505530

[jev270300-bib-0054] McKiernan, J. , M. J. Donovan , V. O'Neill , et al. 2016. “A Novel Urine Exosome Gene Expression Assay to Predict High‐Grade Prostate Cancer at Initial Biopsy.” JAMA oncology 2, no. 7: 882–889. 10.1001/jamaoncol.2016.0097. 27032035

[jev270300-bib-0055] Mei, R. , Z. Wan , C. Yang , et al. 2024. “Advances and Clinical Challenges of Mesenchymal Stem Cell Therapy.” Frontiers in immunology 15: 1421854. 10.3389/fimmu.2024.1421854. 39100671 PMC11294097

[jev270300-bib-0056] Miyazaki‐Anzai, S. , M. Masuda , A. L. Keenan , Y. Shiozaki , J. G. Miranda , and M. Miyazaki . 2024. “Activation of the IKK2/NF‐κB Pathway in VSMCs Inhibits Calcified Vascular Stiffness in CKD.” JCI Insight 9, no. 7: e174977. 10.1172/jci.insight.174977. PMC11128211 38470493

[jev270300-bib-0057] Mizenko, R. R. , M. Feaver , B. T. Bozkurt , et al. 2024. “A Critical Systematic Review of Extracellular Vesicle Clinical Trials.” Journal of Extracellular Vesicles 13, no. 10: e12510. 10.1002/jev2.12510. 39330928 PMC11428870

[jev270300-bib-0058] Musa, R. , P. Rout , and A. Qurie . 2025. “Lupus Nephritis.” In StatPearls. StatPearls Publishing. http://www.ncbi.nlm.nih.gov/books/NBK499817/. 29762992

[jev270300-bib-0059] Nassar, W. , M. El‐Ansary , D. Sabry , et al. 2016. “Umbilical Cord Mesenchymal Stem Cells Derived Extracellular Vesicles Can Safely Ameliorate the Progression of Chronic Kidney Diseases.” Biomaterials Research 20: 21. 10.1186/s40824-016-0068-0. 27499886 PMC4974791

[jev270300-bib-0060] Nieuwland, R. , and P. R. M. Siljander . 2024. “A Beginner's Guide to Study Extracellular Vesicles in Human Blood Plasma and Serum.” Journal of Extracellular Vesicles 13, no. 1: e12400. 10.1002/jev2.12400. 38193375 PMC10775135

[jev270300-bib-0061] Noda, P. , A. L. R. Francini , F. Teles , et al. 2025. “Extracellular Vesicles (EVs) Derived From Mesenchymal Stem Cells (MSCs) as Adjuvants in the Treatment of Chronic Kidney Disease (CKD).” Cells 14, no. 6: 434. 10.3390/cells14060434. 40136683 PMC11941753

[jev270300-bib-0062] Oshikawa‐Hori, S. , N. Yokota‐Ikeda , H. Sonoda , and M. Ikeda . 2019. “Urinary Extracellular Vesicular Release of Aquaporins in Patients With Renal Transplantation.” BMC Nephrology 20, no. 1: 216. 10.1186/s12882-019-1398-7. 31185935 PMC6580655

[jev270300-bib-0063] Pan, B. , J. Qin , X. Liu , et al. 2019. “Identification of Serum Exosomal Hsa‐Circ‐0004771 as a Novel Diagnostic Biomarker of Colorectal Cancer.” Frontiers in Genetics 10: 1096. 10.3389/fgene.2019.01096. 31737058 PMC6838203

[jev270300-bib-0064] Ren, Y. , Y. Chen , X. Zheng , et al. 2020. “Human Amniotic Epithelial Cells Ameliorate Kidney Damage in Ischemia‐Reperfusion Mouse Model of Acute Kidney Injury.” Stem Cell Research & Therapy 11, no. 1: 410. 10.1186/s13287-020-01917-y. 32967729 PMC7510147

[jev270300-bib-0065] Rhim, W. K. , J. Woo , J. Y. Kim , et al. 2025. “Multiplexed PLGA Scaffolds With Nitric Oxide‐Releasing Zinc Oxide and Melatonin‐Modulated Extracellular Vesicles for Severe Chronic Kidney Disease.” Journal of Advanced Research 69: 75–89. 10.1016/j.jare.2024.03.018. 38537702 PMC11954823

[jev270300-bib-0066] Rogers, M. A. , F. Buffolo , F. Schlotter , et al. 2020. “Annexin A1–Dependent Tethering Promotes Extracellular Vesicle Aggregation Revealed With Single–Extracellular Vesicle Analysis.” Science Advances 6, no. 38: eabb1244. 10.1126/sciadv.abb1244. 32938681 PMC7494353

[jev270300-bib-0067] Rosenberg, M. L. , A. Yaneff , G. M. Ferradás , et al. 2023. “Total and Extracellular Vesicle cAMP Contents in Urine Are Associated With Autosomal Dominant Polycystic Kidney Disease (ADPKD) Progression.” Life 13, no. 9: 1817. 10.3390/life13091817. 37763221 PMC10532713

[jev270300-bib-0068] Sakurai, A. , H. Ono , A. Ochi , et al. 2019. “Involvement of Elf3 on Smad3 Activation‐Dependent Injuries in Podocytes and Excretion of Urinary Exosome in Diabetic Nephropathy.” PLoS ONE 14, no. 5: e0216788. 10.1371/journal.pone.0216788. 31150422 PMC6544199

[jev270300-bib-0069] Sedrakyan, S. , V. Villani , S. Da Sacco , et al. 2017. “Amniotic Fluid Stem Cell‐Derived Vesicles Protect From VEGF‐Induced Endothelial Damage.” Scientific Reports 7, no. 1: 16875. 10.1038/s41598-017-17061-2. 29203902 PMC5715019

[jev270300-bib-0070] Sei, H. , N. Hirade , K. Kamiya , et al. 2024. “Isocitrate Dehydrogenase 1 Upregulation in Urinary Extracellular Vesicles From Proximal Tubules of Type 2 Diabetic Rats.” FASEB Journal: Official Publication of the Federation of American Societies for Experimental Biology 38, no. 10: e23688. 10.1096/fj.202400371R. 38780519

[jev270300-bib-0071] Solé, C. , T. Moliné , M. Vidal , J. Ordi‐Ros , and J. Cortés‐Hernández . 2019. “An Exosomal Urinary miRNA Signature for Early Diagnosis of Renal Fibrosis in Lupus Nephritis.” Cells 8, no. 8: 773. 10.3390/cells8080773. 31349698 PMC6721515

[jev270300-bib-0072] Svenningsen, P. , R. Sabaratnam , and B. L. Jensen . 2020. “Urinary Extracellular Vesicles: Origin, Role as Intercellular Messengers and Biomarkers; Efficient Sorting and Potential Treatment Options.” Acta Physiologica 228, no. 1: e13346. 10.1111/apha.13346. 31334916

[jev270300-bib-0073] Tachibana, S. , M. Iyoda , T. Suzuki , et al. 2019. “Serum Uromodulin Is Associated With the Severity of Clinicopathological Findings in ANCA‐Associated Glomerulonephritis.” PLoS ONE 14, no. 11: e0224690. 10.1371/journal.pone.0224690. 31725735 PMC6855443

[jev270300-bib-0074] Tan, L. , H. Zhou , Z. Lai , et al. 2025. “Brain Peptides Modified Exosome‐Mediated Drug Delivery System for Adriamycin‐Induced Nephropathy Treatment.” Nanomedicine: Nanotechnology, Biology and Medicine 66: 102819. 10.1016/j.nano.2025.102819. 40174740

[jev270300-bib-0075] Upson, S. , M. Selesky , M. Greig , et al. “Temporal Dynamics of Urinary Extracellular Vesicle Excretion and Cargo in Healthy Subjects Over 24 Hours.” Preprint, bioRxiv, June 23. 10.1101/2025.06.17.660244.

[jev270300-bib-0076] Usman, W. M. , T. C. Pham , Y. Y. Kwok , et al. 2018. “Efficient RNA Drug Delivery Using Red Blood Cell Extracellular Vesicles.” Nature Communications 9, no. 1: 2359. 10.1038/s41467-018-04791-8. PMC6004015 29907766

[jev270300-bib-0077] Wang, Q. , Z. Shi , X. Xing , et al. 2020. “Matrix Remodeling‐Associated Protein 5 in Urinary Exosomes as a Potential Novel Marker of Obstructive Nephropathy in Children With Ureteropelvic Junction Obstruction.” Frontiers in Pediatrics 8: 504. 10.3389/fped.2020.00504. 32984216 PMC7477104

[jev270300-bib-0078] Wang, Y. , X. Lu , J. He , and W. Zhao . 2015. “Influence of Erythropoietin on Microvesicles Derived From Mesenchymal Stem Cells Protecting Renal Function of Chronic Kidney Disease.” Stem Cell Research & Therapy 6, no. 1: 100. 10.1186/s13287-015-0095-0. 25998259 PMC4469245

[jev270300-bib-0079] Welsh, J. A. , D. C. I. Goberdhan , L. O'Driscoll , et al. 2024. “Minimal Information for Studies of Extracellular Vesicles (MISEV2023): From Basic to Advanced Approaches.” Journal of Extracellular Vesicles 13, no. 2: e12404. 10.1002/jev2.12404. 38326288 PMC10850029

[jev270300-bib-0080] Wu, W. , X. Wu , Z. Cheng , Z. Yang , M. Lu , and J. Cheng . 2022. “Differentially Expressed MicroRNAs in Peritoneal Dialysis Effluent‐Derived Exosomes From the Patients With Ultrafiltration Failure.” Genetical Research 2022: 2276175. 10.1155/2022/2276175. PMC9452989 36101746

[jev270300-bib-0081] Xiao, Z. , Y. Wang , Y. Chen , et al. 2025. “Exosomes Derived From TREM‐2 Knocked‐out Macrophages Alleviated Renal Fibrosis via HSPa1b/AKT Pathway.” American Journal of Physiology. Renal Physiology 328, no. 1: F131–F151. 10.1152/ajprenal.00219.2024. 39657110

[jev270300-bib-0082] Xie, Y. , Y. Jia , X. Cuihua , F. Hu , M. Xue , and Y. Xue . 2017. “Urinary Exosomal MicroRNA Profiling in Incipient Type 2 Diabetic Kidney Disease.” Journal of Diabetes Research 2017: 6978984. 10.1155/2017/6978984. 29038788 PMC5605810

[jev270300-bib-0083] Yamamoto, C. M. , T. Murakami , M. L. Oakes , et al. 2018. “Uromodulin mRNA From Urinary Extracellular Vesicles Correlate to Kidney Function Decline in Type 2 Diabetes Mellitus.” American Journal of Nephrology 47, no. 5: 283–291. 10.1159/000489129. 29779026

[jev270300-bib-0084] Yan, J. , H. Xiao , X. Zhou , et al. 2023. “Engineered Exosomes Reprogram Gli1(+) Cells in Vivo to Prevent Calcification of Vascular Grafts and Autologous Pathological Vessels.” Science Advances 9, no. 29: eadf7858. 10.1126/sciadv.adf7858. 37478186 PMC10361604

[jev270300-bib-0085] Yang, C. , Y. Xue , Y. Duan , C. Mao , and M. Wan . 2024. “Extracellular Vesicles and Their Engineering Strategies, Delivery Systems, and Biomedical Applications.” Journal of Controlled Release 365: 1089–1123. 10.1016/j.jconrel.2023.11.057. 38065416

[jev270300-bib-0086] Zang, J. , A. P. Maxwell , D. A. Simpson , and G. J. McKay . 2019. “Differential Expression of Urinary Exosomal MicroRNAs miR‐21‐5p and miR‐30b‐5p in Individuals With Diabetic Kidney Disease.” Scientific Reports 9, no. 1: 10900. 10.1038/s41598-019-47504-x. 31358876 PMC6662907

[jev270300-bib-0087] Zarovni, N. , and K. Glebov . 2026. “Exosomes': the Hype, the Chasm, and Beyond.” Journal of Extracellular Biology 5, no. 2: e70113. 10.1002/jex2.70113. 41625178 PMC12857696

[jev270300-bib-0088] Zeng, M. , J. Liu , W. Yang , et al. 2019. “Identification of Key Biomarkers in Diabetic Nephropathy via Bioinformatic Analysis.” Journal of Cellular Biochemistry 120, no. 5: 8676–8688. 10.1002/jcb.28155. 30485525

[jev270300-bib-0089] Zhang, C. , L. Cai , M. Ma , X. Xie , J. Wang , and Y. Zhang . 2025. “Hypoxia‐Treated Adipose Mesenchymal Stem Cells Derived Exosomes Enhance the Therapeutic Effects on Unilateral Ureteral Obstruction Mice.” Pharmacology 110, no. 3: 165–177. 10.1159/000542609. 39561719 PMC13200829

[jev270300-bib-0090] Zhang, L. H. , X. Y. Zhu , A. Eirin , et al. 2019. “Early Podocyte Injury and Elevated Levels of Urinary Podocyte‐Derived Extracellular Vesicles in swine With Metabolic Syndrome: Role of Podocyte Mitochondria.” American Journal of Physiology. Renal Physiology 317, no. 7: F12–F22. 10.1152/ajprenal.00399.2018. 31042059 PMC6692726

[jev270300-bib-0091] Zhang, X. , J. Zhao , R. Ge , et al. 2025. “Arg‐Gly‐Asp Engineered Mesenchymal Stem Cells as Targeted Nanotherapeutics Against Kidney Fibrosis by Modulating m6A.” Acta Biomaterialia 198: 85–101. 10.1016/j.actbio.2025.03.042. 40158765

[jev270300-bib-0092] Zhang, Z. Y. , Y. P. Hou , X. Y. Zou , et al. 2020. “Oct‐4 Enhanced the Therapeutic Effects of Mesenchymal Stem Cell‐Derived Extracellular Vesicles in Acute Kidney Injury.” Kidney & Blood Pressure Research 45, no. 1: 95–108. 10.1159/000504368. 31927554

[jev270300-bib-0093] Zhao, M. , S. Liu , Y. Wang , et al. 2025. “In Vivo Reprogramming of Tissue‐Derived Extracellular Vesicles for Treating Chronic Tissue Injury Through Metabolic Engineering.” Advanced Science (Weinheim, Baden‐Württemberg, Germany) 12: 2415556. 10.1002/advs.202415556. 40162496 PMC12140305

[jev270300-bib-0094] Zhao, S. , W. Li , W. Yu , et al. 2021. “Exosomal miR‐21 From Tubular Cells Contributes to Renal Fibrosis by Activating Fibroblasts via Targeting PTEN in Obstructed Kidneys.” Theranostics 11, no. 18: 8660–8673. 10.7150/thno.62820. 34522205 PMC8419054

[jev270300-bib-0095] Zhao, Y. , X. Zhu , L. Zhang , et al. 2020. “Mesenchymal Stem/Stromal Cells and Their Extracellular Vesicle Progeny Decrease Injury in Poststenotic Swine Kidney through Different Mechanisms.” Stem Cells and Development 29, no. 18: 1190–1200. 10.1089/scd.2020.0030. 32657229 PMC7482134

[jev270300-bib-0096] Zheng, Y. , H. Wang , X. Li , J. Xie , J. Fan , and S. Ren . 2024. “Extracellular Vesicles in Chronic Kidney Disease: Diagnostic and Therapeutic Roles.” Frontiers in pharmacology 15: 1371874. 10.3389/fphar.2024.1371874. 38545551 PMC10965796

[jev270300-bib-0097] Zhou, L. T. , S. Qiu , L. L. Lv , et al. 2018. “Integrative Bioinformatics Analysis Provides Insight Into the Molecular Mechanisms of Chronic Kidney Disease.” Kidney & Blood Pressure Research 43, no. 2: 568–581. 10.1159/000488830. 29642064

[jev270300-bib-0098] Zhou, R. , Y. Zhen , H. Ma , et al. 2025. “Transcriptome Profiling of Serum Exosomes by RNA‐Seq Reveals Lipid Metabolic Changes as a Potential Biomarker for Evaluation of Roxadustat Treatment of Chronic Kidney Diseases.” Molecular Omics 21, no. 3: 240–249. 10.1039/d4mo00025k. 40094436

[jev270300-bib-0099] Zhou, S. , J. Fang , M. Hu , et al. 2021. “Determining the Influence of High Glucose on Exosomal lncRNAs, mRNAs, circRNAs and miRNAs Derived From Human Renal Tubular Epithelial Cells.” Aging 13, no. 6: 8467–8480. 10.18632/aging.202656. 33714195 PMC8034913

[jev270300-bib-0100] Zhou, X. , S. Zhao , W. Li , et al. 2021. “Tubular Cell‐Derived Exosomal miR‐150‐5p Contributes to Renal Fibrosis Following Unilateral Ischemia‐Reperfusion Injury by Activating Fibroblast in Vitro and in Vivo.” International Journal of Biological Sciences 17, no. 14: 4021–4033. 10.7150/ijbs.62478. 34671216 PMC8495396

[jev270300-bib-0101] Zhou, Y. , L. Fang , Y. Yu , et al. 2016. “Erythropoietin Protects the Tubular Basement Membrane by Promoting the Bone Marrow to Release Extracellular Vesicles Containing tPA‐Targeting miR‐144.” American Journal of Physiology. Renal Physiology 310, no. 1: F27–40. 10.1152/ajprenal.00303.2015. 26469975

[jev270300-bib-0102] Zhou, Y. , S. Liu , M. Zhao , et al. 2019. “Injectable Extracellular Vesicle‐Released Self‐Assembling Peptide Nanofiber Hydrogel as an Enhanced Cell‐Free Therapy for Tissue Regeneration.” Journal of Controlled Release: Official Journal of the Controlled Release Society 316: 93–104. 10.1016/j.jconrel.2019.11.003. 31704110

